# HAT1 functions as a lactyltransferase and mediates RPA1 lactylation to promote DNA repair and radioresistance in lung adenocarcinoma

**DOI:** 10.1038/s41419-025-08113-x

**Published:** 2025-11-21

**Authors:** Jiang He, Tangmin Lai, Yuzu Zhao, Zhiying Zhou, Liu Zhou, Dan Tao, Haonan Yang, Nan Li, Yu He, Shuheng Yang, Zheng Tang, Siwei Zeng, Erha Munai, Yanchen Liu, Yuanyuan Tan, Wei Zhou, Yongzhong Wu

**Affiliations:** 1 College of Bioengineering, Chongqing University; School of Medicine, Chongqing University; Chongqing University Cancer Hospital, Chongqing, China; 2 Radiation Oncology Center, Chongqing University Cancer Hospital, Chongqing, China; 3 Department of Breast Cancer Center, Chongqing University Cancer Hospital, Chongqing, China

**Keywords:** Non-small-cell lung cancer, Non-small-cell lung cancer

## Abstract

Lysine lactylation is a post-translational modification induced by lactate discovered in recent years. Abnormal lysine lactylation contributes to the occurrence and progression of various tumors. However, the mediators and downstream targets of lysine lactylation remain unclear. Here, we report that HAT1 serves as a potential lactyltransferase that can promote homologous recombination and lead to radioresistance by regulating lactylation of RPA1. Lactylation of RPA1 facilitates its binding to single-stranded DNA and MRE11-RAD50-NBS1 (MRN) complexes and promotes homologous recombination. HAT1 knockout inhibits DNA repair in lung adenocarcinoma cells, thereby increasing radiotherapy sensitivity. Interestingly, we also found that K15 auto-lactylation of HAT1 can modulate its lactyltransferase activity. In conclusion, our research reveals that HAT1-regulated RPA1 lactylation plays an important role in homologous recombination and radioresistance, suggesting that HAT1 may become a potential therapeutic target for reversing the radioresistance caused by lactate accumulation in cancer cells.

## Introduction

Most cancers have a metabolic characteristic called the Warburg effect, which allows cancer cells to continue glycolysis and produce large amounts of lactate even in the presence of oxygen [1, 2]. Research shows that targeting the metabolic process of lactate production can inhibit tumor progression [3]. Recent research has revealed that aside from its role in metabolic processes, lactate can also engage in post-translational modifications (PTM), triggering the lactylation alteration of lysine residues within proteins [4]. The latest studies confirm the involvement of lysine lactylation in regulating various biological processes in tumor cells [5–8]. However, the functions and regulatory mechanisms of lactylated proteins are still not fully understood, and further research is needed to elucidate these mechanisms from obscurity.

Lung adenocarcinoma (LUAD) is the most common type of non-small cell lung cancer (NSCLC), and its incidence has gradually increased in recent years [9, 10]. Radiotherapy serves as a critical treatment modality and plays a crucial role in the management of LUAD patients [11]. Radiotherapy kills tumor cells by causing DNA double-strand breaks (DSBs) [12–14]. Currently, two main pathways are responsible for repairing DSBs: non-homologous end joining (NHEJ) and homologous recombination (HR) [12, 15]. NHEJ directly joins the broken DNA ends together. It is simpler and faster than HR, but has the obvious disadvantage of being error-prone and may cause a large number of mutations. NHEJ operates throughout the cell cycle, while HR, requiring homologous templates, predominantly occurs during S and G2 phases. However, HR can precisely repair DSBs, prevent the introduction of mutations, and enhance cell survival [16]. The primary stages of HR involve the end resection of damaged DNA, yielding suitable single-stranded DNA (ssDNA) [17]. The resulting ssDNA is rapidly coated and protected by replication protein A (RPA1), binding of RPA1 prevents ssDNA from wrapping around itself or forming secondary structures. RPA1 is subsequently replaced by RAD51 recombinase to form nucleofilaments, which is critical for recognition of homologous fragments and subsequent steps of the HR process [18]. HR overactivation is the main driver of tumor cell radioresistance [19–21]. Recent studies indicate that lactylation of proteins such as MRE11 and NBS1 is crucial in the HR process [5, 6], offering novel strategies for radiotherapy sensitization. Nonetheless, it remains uncertain whether the lactylation of RPA1 regulates HR efficiency.

Lactylation is the latest type of novel acylation modification [22]. According to current research, histone acetyltransferases, deacetylases, and their readers are also key regulatory factors of the novel acylation modification [22]. Based on existing studies, the lysine acetyltransferase family (KAT family) proteins have been identified as the main writers responsible for lactylation modification [4–6, 22–24]. The first reported lactyltransferase P300 belongs to the KAT family [4]. Subsequent studies have shown that other members of the KAT family, such as CBP [5], KAT5 [6], and KAT8 [23], also exhibit lactyltransferase activity. They can not only catalyze the lactylation of histones, but also regulate the lactylation modification of non-histone proteins. HAT1 is the first member identified in the KAT family [25]. HAT1 can acetylate histones at different sites, such as H4K5, H4K12, H3K9, H3K18, and H3K27 [26]. In addition, studies have shown that it can also acetylate non-histone proteins [27]. Furthermore, recent studies have demonstrated that HAT1 can also regulate lysine succinylation and methacrylation [28, 29]. Although HAT1 was the first histone acetyltransferase discovered, its biological functions are poorly characterized.

In this study, we found that HAT1 is highly expressed in lung adenocarcinoma and impairs patient survival. HAT1 knockout significantly reduces protein lactylation levels globally, suggesting its potential role as a lactyltransferase. Further results indicate that HAT1 promotes the lactylation of RPA1, which plays a crucial role in regulating its ability to bind to ssDNA and HR complexes. Interestingly, we found that HAT1 auto-lactylation at K15 is required for its lactyltransferase activity. HAT1 knockout can inhibit HR and increase radiosensitivity. Taken together, our findings indicate that HAT1 promotes HR and radioresistance by facilitating RPA1 lactylation, and targeting HAT1 may be an effective strategy to reverse radioresistance induced by lactate.

## Results

### HAT1 is upregulated in LUAD and correlated with poor prognosis

**Fig. 1:**
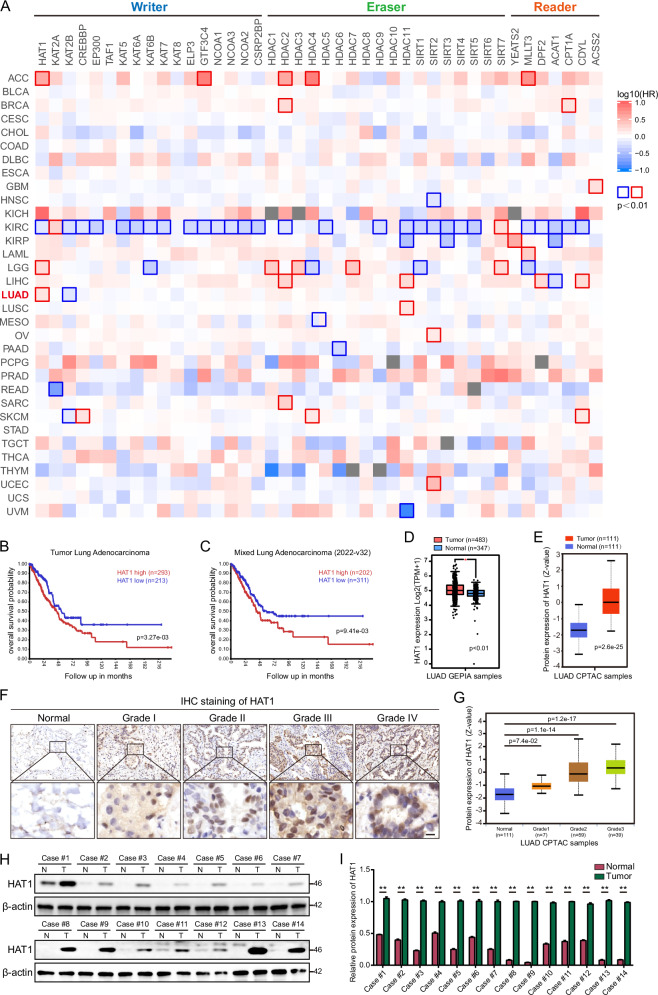
HAT1 is upregulated in LUAD and correlated with poor prognosis. **A** Survival map analysis using the GEPIA database with a median cutoff. ACC (Adrenocortical carcinoma), BLCA (Bladder Urothelial Carcinoma), BRCA (Breast invasive carcinoma), CESC (Cervical squamous cell carcinoma and endocervical adenocarcinoma), CHOL(Cholangio carcinoma), COAD (Colon adenocarcinoma), DLBC (Lymphoid Neoplasm Diffuse Large B-cell Lymphoma), ESCA (Esophageal carcinoma), GBM (Glioblastoma multiforme), HNSC (Head and Neck squamous cell carcinoma), KICH (Kidney Chromophobe), KIRC (Kidney renal clear cell carcinoma), KIRP (Kidney renal papillary cell carcinoma), LAML (Acute Myeloid Leukemia), LGG(Brain Lower Grade Glioma), LIHC (Liver hepatocellular carcinoma), LUAD (Lung adenocarcinoma), LUSC (Lung squamous cell carcinoma), MESO (Mesothelioma), OV (Ovarian serous cystadenocarcinoma), PAAD (Pancreatic adenocarcinoma), PCPG (Pheochromocytoma and Paraganglioma), PRAD (Prostate adenocarcinoma), READ (Rectum adenocarcinoma), SARC (Sarcoma), SKCM (Skin Cutaneous Melanoma), STAD (Stomach adenocarcinoma), TGCT (Testicular Germ Cell Tumors), THCA (Thyroid carcinoma), THYM (Thymoma), UCEC (Uterine Corpus Endometrial Carcinoma), UCS (Uterine Carcinosarcoma), UVM (Uveal Melanoma). **B** Kaplan-Meier analysis of patients with high or low expression of HAT1 using the Tumor Lung Adenocarcinoma data set. **C** Kaplan-Meier analysis of patients with high or low expression of HAT1 using Mixed Lung Adenocarcinoma data set. **D** Analysis of the expression of HAT1 in LUAD GEPIA samples. **E** Analysis of the expression of HAT1 in LUAD CPTAC samples. **F** HAT1 IHC staining in normal tissues and in tumor tissues of different grades, including Grade Ⅰ, Grade Ⅱ, Grade Ⅲ and Grade Ⅳ (scale bar = 10 μm). **G** Analysis of the expression of HAT1 in normal tissues and in tumor tissues of different grades including Grade Ⅰ, Grade Ⅱ and Grade Ⅲ using LUAD CPTAC data set. **H** The expression of HAT1 in 14 pairs of normal and tumor tissues. (**I**) Relative protein expression of HAT1 in panel H. Data were analyzed using two-way analysis of variance (ANOVA), **P < 0.01.

We performed survival map analysis on the currently known main lysine acetyltransferases (writer), deacetylases (eraser), and readers using the GEPIA database [30], and the results revealed that high expression of HAT1 is significantly associated with poor prognosis in LUAD patients (Fig. 1A). Next, using Kaplan-Meier analysis in R2: Genomics Analysis and Visualization Platform, the correlation between HAT1 expression and the prognosis of human LUAD was analyzed. We found that patients with high expression of HAT1 showed poor survival (Fig. 1B, C), suggesting that HAT1 might act as a candidate prognostic biomarker for predicting therapeutic outcomes. In evaluating the expression of HAT1 in LUAD patients using GEPIA and CPTAC databases, we found that the level of HAT1 was elevated in tumor samples compared with normal tissues (Fig. 1D, E). Furthermore, immunohistochemistry (IHC) staining was conducted to detect the expression of HAT1 in LUAD samples, and we observed that HAT1 was highly expressed in LUAD tissues in a grade-dependent manner (Fig. 1F). We next analyzed the protein expression of HAT1 in LUAD patients using the CPTAC database, and consistent with IHC staining, the level of HAT1 was higher in LUAD patients in a grade-dependent manner (Fig. 1G). We further examined HAT1 in 14 pairs of normal and tumor tissues, and HAT1 was upregulated in the tumor tissues (Fig. 1H, I). These results indicate that HAT1 might be a potential oncogene in LUAD.

### HAT1 is required for the lactylation of LUAD cells

**Fig. 2:**
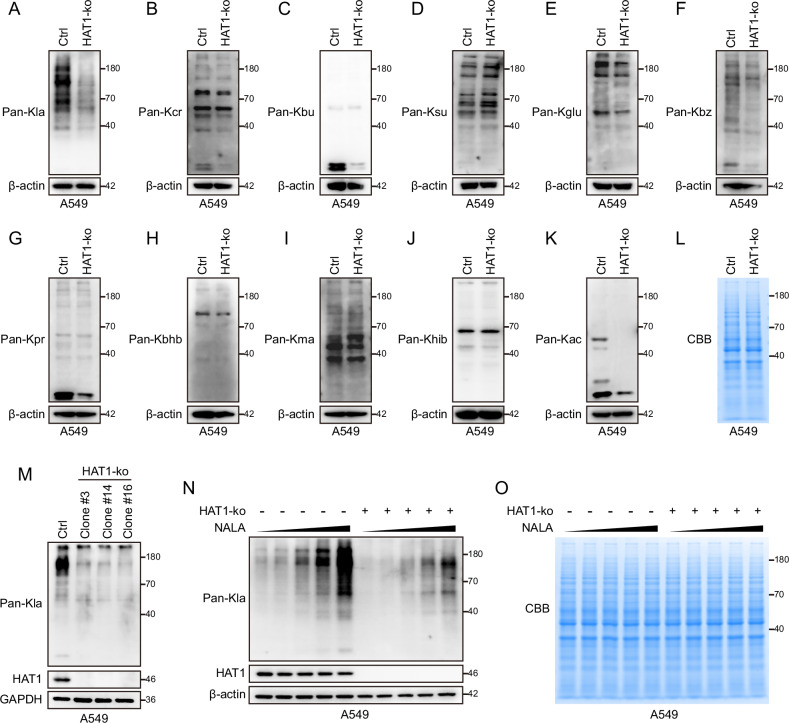
HAT1 regulate lactylation in LUAD cells. **A** The level of pan L-Lactyl Lysine (Pan-Kla) in HAT1-KO Cells. **B** The expression of pan Crotonyllysine (Pan-Kcr) in HAT1-KO Cells. **C** The level of pan Butyryllysine (Pan-Kbu) in HAT1-KO Cells. **D** The expression of global Succinyllysine (Pan-Ksu) in HAT1-KO Cells. **E** The level of global Glutaryllysine (Pan-Kglu) in HAT1-KO Cells. **F** The expression of global Benzoyllysine (Pan-Kbz) in HAT1-KO Cells. **G** The level of pan Propionyllysine (Pan-Kpr) in HAT1-KO Cells. **H** The expression of global β-Hydroxybutyryllysine (Pan-Kbhb) in HAT1-KO Cells. **I** The level of global Malonyllysine (Pan-Kma) in HAT1-KO Cells. **J** The level of pan 2-Hydroxyisobutyryllysine (Pan-Khib) in HAT1-KO Cells. **K** The expression of global Acetyllysine (Pan-Kac) in HAT1-KO Cells. **L** Coomassie brilliant blue staining of total proteins in control cells and in HAT1-KO cells. **M** The expression of pan-Kla, HAT1 and GAPDH in different monoclonal cells depleting of HAT1. **N** NALA induced pan-Kla in HAT1-KO cells. **O** Coomassie brilliant blue staining of total proteins induced by NALA in HAT1-KO cells.

The novel acylation modifications play important roles in signaling transduction and in various physiological functions [22]. Misregulation of acylation can lead to cancer [31–34]. To further investigate the role of HAT1 in LUAD, HAT1-knockout (HAT1-KO) A549 and H1299 cells were established (Supplementary Fig. 1A-D). Since HAT1 is a KAT family acetyltransferase, we speculate that HAT1 might promote LUAD by regulating some other types of novel acylation modifications. To test this hypothesis, several types of acylation were assessed in control cells and HAT1-KO cells. The results showed that only L-Lactyl Lysine (Fig. 2A and Supplementary Fig. 2A) and Acetyllysine (Fig. 2K and Supplementary Fig. 2K) were significantly decreased in HAT1-KO cells, the other 9 types of acylation, including Crotonyllysine (Fig. 2B and Supplementary Fig. 2B), Butyryllysine (Fig. 2C and Supplementary Fig. 2C), Succinyllysine (Fig. 2D and Supplementary Fig. 2D), Glutaryllysine (Fig. 2E and Supplementary Fig. 2E), Benzoyllysine (Fig. 2F and Supplementary Fig. 2F), Propionyllysine (Fig. 2G and Supplementary Fig. 2 G), β-Hydroxybutyryllysine (Fig. 2H and Supplementary Fig. 2H), Malonyllysine (Fig. 2I and Supplementary Fig. 2I) and 2-Hydroxyisobutyryllysine (Fig. 2J and Supplementary Fig. 2J), were not regulated by HAT1. Coomassie brilliant blue staining indicated that the total proteins between control cells and HAT1-KO cells were equal (Fig. 2L and Supplementary Fig. 2L). For the first time, this study reveals HAT1 as a regulator of the global lactylation in LUAD cells. Global lysine lactylation was further assessed in different HAT1-KO monoclonal LUAD cells, and the level of lysine lactylation was significantly decreased in each HAT1-KO monoclonal A549 cell (Fig. 2M). Furthermore, we treated A549 cells with a gradient concentration of sodium lactate (NALA), which induced the lactylation of proteins [4, 5]. Consistent with the results mentioned above, lysine lactylation was significantly decreased in HAT1-KO A549 cells (Fig. 2N). Coomassie brilliant blue staining indicated that the total proteins between control cells and HAT1-KO cells were equal (Fig. 2O).

### HAT1 promotes DNA damage repair (DDR) in LUAD cells

**Fig. 3:**
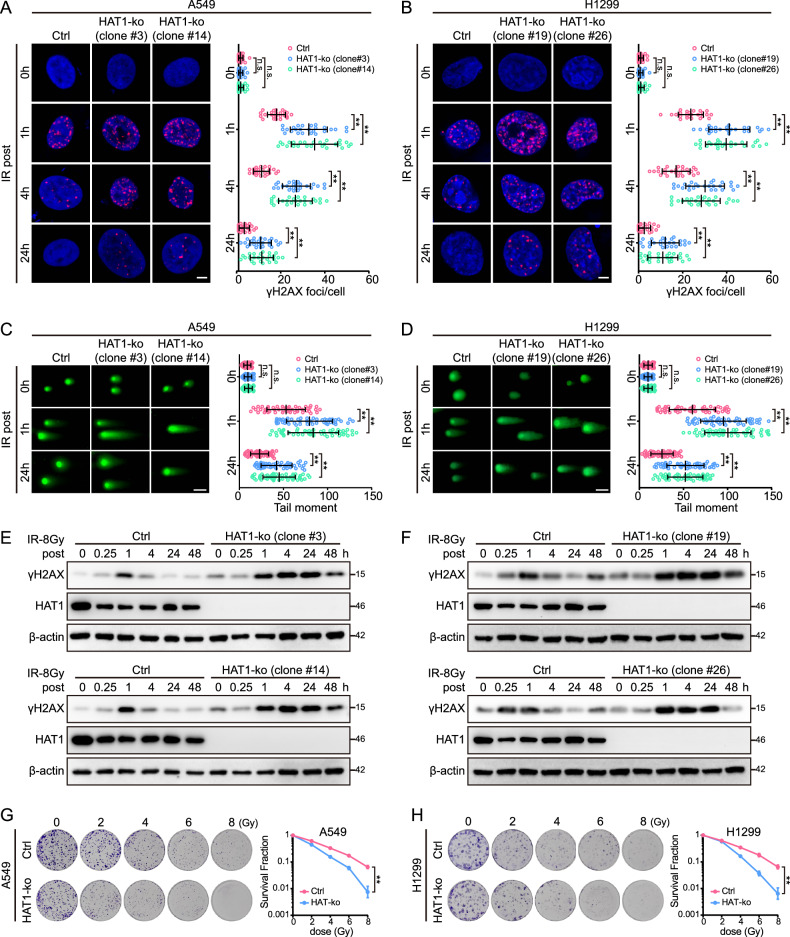
HAT1 promotes DDR in LUAD cells. **A** The IR-induced foci of γ-H2AX in A549 cells abolishing of HAT1 (scale bar = 5 μm; *n* = 25). **B** The IR-induced foci of γ-H2AX in H1299 cells depleting of HAT1 (scale bar = 5 μm; *n* = 25). **C** The IR-induced tailed DNA in HAT1-KO A549 cells (scale bar=10 μm; *n* = 50). **D** The tailed DNA in H1299 cells depleting of HAT1 (scale bar = 10 μm; *n* = 50). **E** The expression of γ-H2AX, HAT1 and β-actin in monoclonal A549 cells depleting of HAT1 (clone #3 and clone #14). **F** The expression of γ-H2AX, HAT1 and β-actin in monoclonal H1299 cells depleting of HAT1 (clone #19 and clone #26). **G** The clonogenic ability of A549 cells treated with IR. **H** The colony-forming ability of H1299 cells treated with IR. All data were analyzed using two-way analysis of variance (ANOVA), **P < 0.01.

It has been reported that lactylation acts as a critical mechanism for DNA repair [5, 6, 35]. Ionizing radiation (IR) exposure causes DNA double-strand breaks (DSBs), which are the most deleterious form of DNA damage [15]. To investigate the function of HAT1 in DDR, we first examined the IR-induced foci of γ-H2AX, which is a classic marker for DDR [36]. The persistence of γ-H2AX foci was prolonged in HAT1-KO A549 and H1299 cells compared to control cells (Fig. 3A, B), indicating that depletion of HAT1 impaired the DDR of LUAD cells. Comet assays were further conducted and revealed that the comet tail distance was significantly increased in HAT1-KO cells compared to control cells (Fig. 3C, D). Next, the expression of γ-H2AX after IR was detected by immunoblot, and the expression of γ-H2AX increased significantly in HAT-KO A549 and H1299 cells (Fig. 3E, F). We next performed colony formation assays to evaluate the role of HAT1 in radiotherapy sensitivity, and found that depletion of HAT1 inhibits colony survival in LUAD cells (Fig. 3G, H). Taken together, these results suggest that HAT1 might play a key role in DDR.

### HAT1 promotes HR-mediated DDR in LUAD cells

**Fig. 4:**
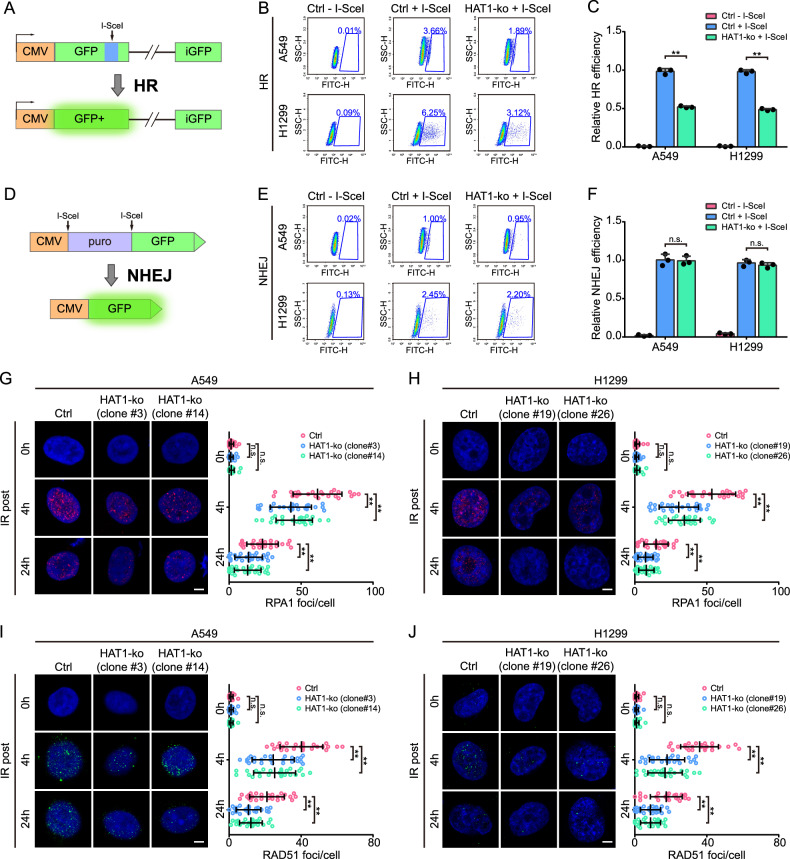
HAT1 promotes HR-mediated DDR in LUAD cells. **A** Schematic diagram of HR reporter systems. **B** GFP-positive cells determined by HR reporter systems using flow cytometry. **C** Relative HR efficiency of control LUAD cells and HAT1-KO LUAD cells. **D** Schematic diagram of NHEJ reporter systems. **E** GFP-positive cells determined by NHEJ reporter systems using flow cytometry. **F** Relative NHEJ efficiency of control LUAD cells and HAT1-KO LUAD cells. **G** The IR-induced foci of RPA1 in A549 cells, abolishing of HAT1 (scale bar = 5 μm; *n* = 25). **H** The IR-induced foci of RPA1 in H1299 cells depleted of HAT1 (scale bar = 5 μm; *n* = 25). **I** The IR-induced foci of RAD51in A549 cells, abolishing of HAT1 (scale bar = 5 μm; *n* = 25). **J** The IR-induced foci of RAD51 in H1299 cells depleting of HAT1 (scale bar = 5 μm; *n* = 25). All data were analyzed using two-way analysis of variance (ANOVA), **P < 0.01.

DSBs are repaired through two primary mechanisms: homologous recombination (HR) and nonhomologous end joining (NHEJ) [12]. To further investigate the role of HAT1 in DDR, HR efficiency and NHEJ efficiency were evaluated using HR (Fig. 4A) and NHEJ (Fig. 4D) reporter assay, respectively. Compared with control cells, the HR efficiency decreased in HAT1-KO cells (Fig. 4B, C). On the contrary, depletion of HAT1 did not affect the NHEJ efficiency of LUAD cells (Fig. 4E, F). These results indicate that HAT1 promotes DDR through HR rather than NHEJ. Furthermore, we detected the two key regulators of HR, RPA1 and RAD51, through immunofluorescence. The persistence of RPA1 foci was reduced in HAT1-KO A549 and H1299 cells compared to control cells (Fig. 4G, H). Consistent with the results shown above, the formation of RAD51 foci was also impaired in HAT1-KO A549 and H1299 cells compared to control cells (Fig. 4I, J). These results illustrate that HAT1 promotes HR-mediated DDR in LUAD cells.

### HAT1 positively regulates lactylation of RPA1 in LUAD cells

**Fig. 5:**
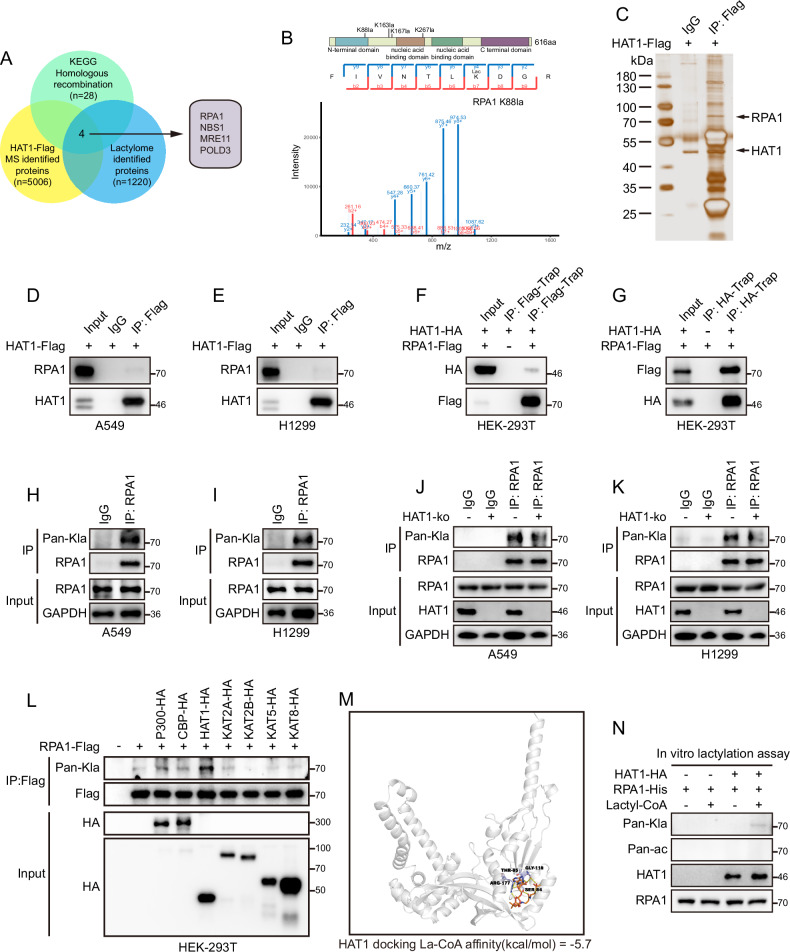
HAT1 mediates lactylation of RPA1 in LUAD cells. **A** Intersection among lysine-lactylated substrates, HAT1-interacted proteins and the gene set of 28 HR proteins. **B** Secondary mass spectrometry map of RPA1 K88 lactylation modification. **C** Sliver staining of proteins interacted with HAT1. **D** Interaction of HAT1 with RPA1 in A549 cells. **E** Interaction of HAT1 with RPA1 in H1299 cells. **F** Interaction of exogenous HAT1 with exogenous RPA1 in 293 T cells detected by Flag-Trap. **G** Interaction of exogenous HAT1 with exogenous RPA1 in 293 T cells detected by HA-Trap. **H** Validation of RPA1 lactylation in A549 cells. **I** Confirmation of RPA1 lactylation in H1299 cells. **J** Detection of lactylated RPA1 in A549 cells depleting of HAT1. **K** Detection of lactylated RPA1 in H1299 cells depleting of HAT1. **L** Detection of lactylated RPA1 in HEK-293T cells overexpressed with 7 acetyltransferase of KAT family including P300, CBP, HAT1, KAT2A, KAT2B, KAT5 and KAT8. **M** Schematic diagram of molecular docking between HAT1 and La-CoA. **N** In vitro lactylation assay showing lysine lactyltransferase activity of HAT1 towards RPA1.

As mentioned above, HAT1 is required for the lactylation of LUAD cells. In addition, HAT1 promotes HR-mediated DDR in LUAD cells. Therefore, we speculated that HAT1 promotes HR-mediated DDR by regulating lactylation of LUAD cells. To further verify our hypothesis, 4D label-free lactylation proteomics was performed to explore the profile of lactylated proteins in A549 cells, and 1220 lactylated proteins were identified (Supplementary Table 1). Besides, we conducted high-resolution liquid chromatography-tandem MS (LC-MS/MS) to investigate the HAT1-interacted proteins using Flag beads pulldown and found 5006 proteins that interact with HAT1 (Supplementary Table 2). Next, an intersection among lactylated proteins, HAT1-interacted proteins, and the gene set of 28 HR proteins was analyzed, and the results suggested that RPA1, NBS1, MRE11 and POLD3 are the potential lysine-lactylated substrates of HAT1 that may regulate the HR-mediated DDR in LUAD cells (Fig. 5A). In the core HR complex consisting of RPA1, NBS1, and MRE11, lactylation of NBS1 and MRE11 has been reported to promote HR in recent studies [5, 6]. However, whether lactylated RPA1 regulates HR remains unclear. Using lactylation proteomics, we found that 4 lysine sites (K88, K163, K167 and K267) of RPA1 were lactylated (Fig. 5B and Supplementary Fig. 3A-C). Silver staining was performed and indicated that HAT1 interacts with RPA1 (Fig. 5C). Furthermore, Immunoprecipitation (IP) demonstrated that RPA1 bound to HAT1 in A549 cells (Fig. 5D) as well as in H1299 cells (Fig. 5E). Consistent with these results, exogenous HAT1 interacted with exogenous RPA1 in 293 T cells (Fig. 5F, G). Next, using pan-lactylation antibody, we confirmed that RPA1 is lactylated in A549 cells (Fig. 5H) and in H1299 cells (Fig. 5I). In addition, depletion of HAT1 decreased the lactylation level of RPA1 in A549 cells (Fig. 5J) and H1299 cells (Fig. 5K). To further validate the regulation of HAT1 on the lactylation of RPA1, we overexpressed 7 acetyltransferases of the KAT family, including P300, CBP, HAT1, KAT2A, KAT2B, KAT5 and KAT8, in 293 T cells and found that lactylated RPA1 increased most significantly in HAT1-overexpressing cells (Fig. 5L). Molecular docking further indicated the interaction between HAT1 and Lactyl-CoA (La-CoA) (Fig. 5M). In vitro lactylation assays confirmed that HAT1 mediated the lactylation of RPA1 (Fig. 5N). Taken together, these results indicate that HAT1 binds to RPA1 and regulates its lactylation.

### Lactylation of RPA1 promotes HR

**Fig. 6:**
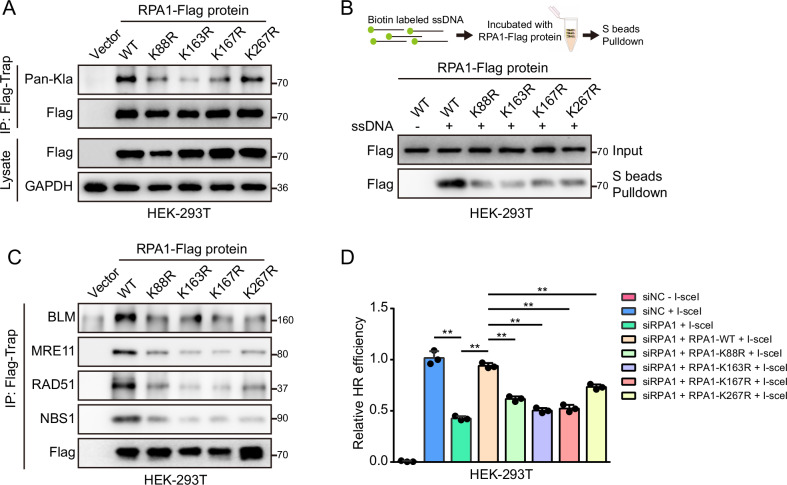
Lactylation of RPA1 on K88, K163, K167, and K267 sites promotes HR. **A** The level of lactylated RPA1 in mutant cells with RPA1 K88R, K163R, K167R, and K267R. **B** The level of RPA1 interacting ssDNA in mutant cells with RPA1 K88R, K163R, K167R and K267R. **C** The level of RPA1 interacted with HR factors, including BLM, MRE11, RAD51, and NBS1, in mutant cells with RPA1 K88R, K163R, K167R, and K267R. **D** Relative HR efficiency in mutant cells with RPA1 K88R, K163R, K167R and K267R. All data were analyzed using two-way analysis of variance (ANOVA), **P < 0.01.

Using 4D label-free lactylation proteomics, as noted above, 4 lysine lactylation sites including K88, K163, K167 and K267 of RPA1 were identified. We then generated site-specific mutations by replacing lysine (K) at positions 88, 163, 167, and 267 of RPA1 with arginine (R). The results indicate that lactylation of RPA1 decreased in mutant cells with RPA1 K88R, K163R, K167R, and K267R (Fig. 6A). Biotin-labeled ssDNA was synthesized to perform a DNA-protein binding assay. Compared with WT-RPA1, the affinity of RPA1 with ssDNA was attenuated in mutant cells (Fig. 6B). Next, we assessed whether the lactylation of RPA1 affected the interaction between RPA1 and other HR factors. 293 T cells transfected with RPA1 K88R, K163R, K167R, and K267R were harvested, and the RPA1-bound HR factors, including BLM, MRE11, RAD51, and NBS1, were detected. RPA1 K88R, K163R, K167R, and K267R mutant cells showed weakened interactions with BLM, MRE11, RAD51, and NBS1, indicating that lactylation of RPA1 is important for its association with other HR factors (Fig. 6C). HR efficiency was further evaluated, and the results reveal that mutations at RPA1 lysine sites including K88, K163, K167, and K267 ultimately inhibit the HR process in 293 T cells (Fig. 6D). Collectively, lactylation of RPA1 on K88, K163, K167 and K267 sites promoted the interaction of RPA1 with ssDNA as well as HR factors and thereby the lactylated RPA1 enhanced HR efficiency.

### Auto-lactylation of HAT1 on K15 is indispensable for its function as a lactylation writer

**Fig. 7:**
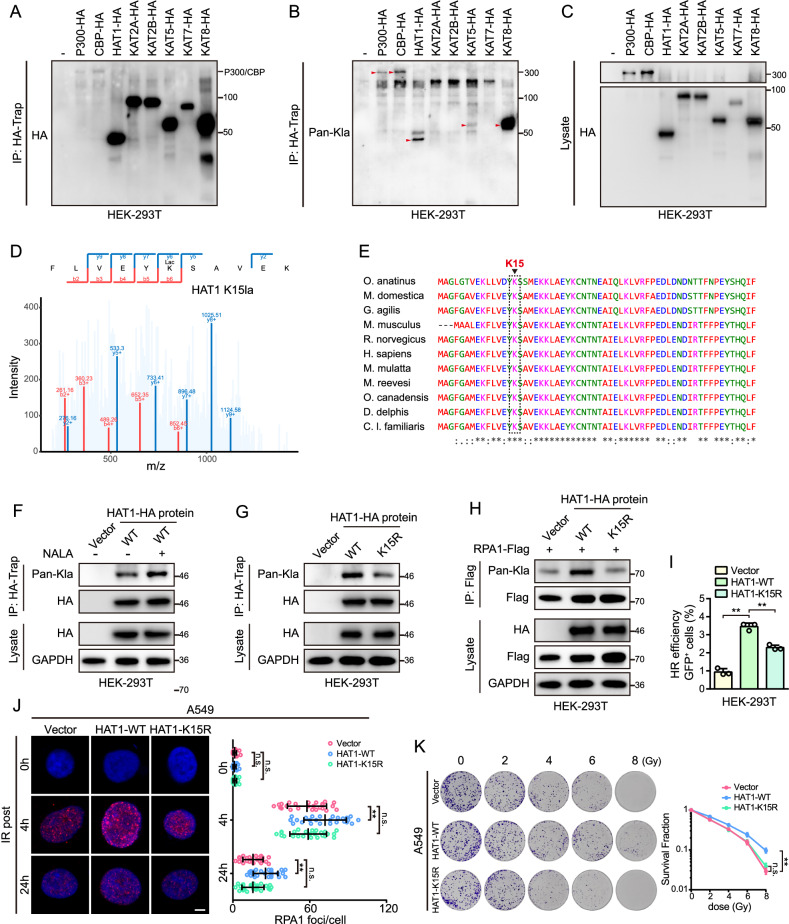
Auto-lactylation of HAT1 on K15 promotes the lactylation of RPA1. **A** Detection of HA-Trap binded acetyltransferase in cells overexpressed 8 acetyltransferase of KAT family, including P300, CBP, HAT1, KAT2A, KAT2B, KAT5, KAT7 and KAT8. **B** The level of lactylated acetyltransferases in cells overexpressed 8 acetyltransferase of KAT family, including P300, CBP, HAT1, KAT2A, KAT2B, KAT5, KAT7, and KAT8 (the red arrows represent the corresponding lactylated proteins). **C** The expression of acetyltransferase in whole cell lysates overexpressed 8 acetyltransferase of KAT family, including P300, CBP, HAT1, KAT2A, KAT2B, KAT5, KAT7, and KAT8. **D** Secondary mass spectrometry map of HAT5 K15 lactylation modification. **E** Sequence alignment of the K15 site of HAT1 in diverse species. **F** The level of NALA-induced lactylated HAT1 in 293 T cells. **G** The expression of lactylated HAT1 in WT cells as well as in mutant cells with HAT1 K15R. **H** The level of lactylated RPA1 in HAT1-WT cells as well as in mutant cells with HAT1-K15R. **I** Relative HR efficiency in WT cells and in mutant cells with HAT1-K15R. **J** The IR-induced foci of RPA1 in A549 cells expressing HAT1-WT proteins or mutant HAT1-K15R (scale bar=5 μm; *n* = 25). **K** The colony-forming ability of A549 cells expressing HAT1-WT proteins or mutant HAT1-K15R. All data were analyzed using two-way analysis of variance (ANOVA), **P < 0.01.

Previous studies have suggested that auto-acetylation is required for the acetyltransferase activity of KAT family proteins [37–39]. To further investigate whether HAT1 exhibits auto-lactylation and whether auto-lactylation modulates its lactyltransferase activity, we analyzed the auto-lactylation of HAT1. Initially, we overexpressed 8 acetyltransferases of the KAT family, including P300, CBP, HAT1, KAT2A, KAT2B, KAT5, KAT7, and KAT8, in 293 T cells and found that P300, CBP, HAT1, KAT5, and KAT8 were lactylated (Fig. 7A–C). Interestingly, of these five proteins, besides HAT1, the other four, including P300 [4], CBP [5], KAT5 [6], and KAT8 [23] have been confirmed to possess lactyltransferase activity. According to lactylation proteomics mentioned above, the K15 site was identified as a HAT1 lactylated site (Fig. 7D). Next, sequence alignment showed that the K15 site of HAT1 is highly conserved in diverse species (Fig. 7E). Furthermore, IP assays indicated that NALA enhanced the lactylation of HAT1 (Fig. 7F). Moreover, replacing lysine (K) at site 15 with arginine (R) (K15R) decreased HAT1 auto-lactylation (Fig. 7G), indicating that HAT1 is lactylated at K15. In addition, the ability of the HAT1-K15R mutant to promote RPA1 lactylation is significantly inhibited compared to the HAT1-WT proteins (Fig. 7H). HR efficiency was further evaluated and revealed that the mutation of HAT1 K15 site attenuated HR efficiency (Fig. 7I). The IR-induced foci of RPA1 increased in HAT1-WT overexpressed cells but not in HAT1-K15R cells (Fig. 7J). Moreover, the mutation of HAT1 K15R inhibited colony survival after IR (Fig. 7K). These results suggest that auto-lactylation of HAT1 enhances its lactyltransferase activity and promotes radioresistance.

### HAT1 enhances lactylation to promote radiotherapy resistance in LUAD

**Fig. 8:**
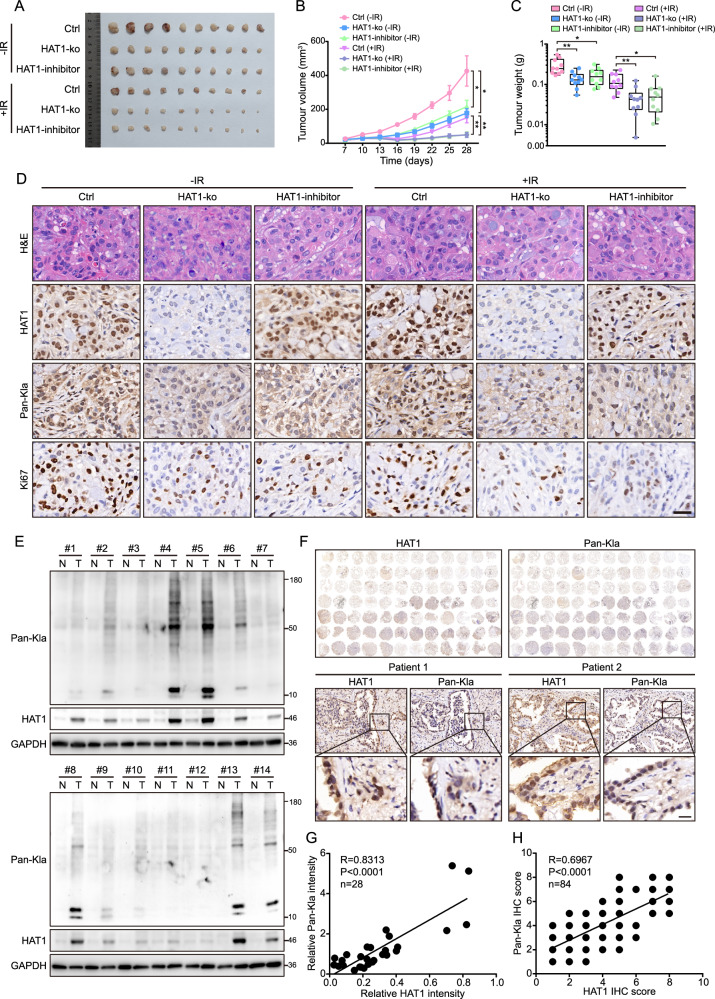
HAT1-mediated lactylation promotes radiotherapy resistance in LUAD. **A** Photograph of tumors from indicated mice, including the control group, HAT1-KO group, and HAT1 inhibitor group with or without IR. **B** The tumor volume of the indicated mice, including the control group, the HAT1-KO group, and the HAT1 inhibitor group with or without IR. **C** The tumor weight of indicated mice, including the control group, HAT1-KO group, and HAT1 inhibitor group with or without IR. **D** H&E staining and IHC staining with Pan-Kla, HAT1, and Ki-67 in tumors from the control group, HAT1-KO group, and HAT1 inhibitor group with or without IR (scale bar = 30 μm). **E** The expression of global lactylated proteins, HAT1, and GAPDH in 14 pairs of normal tissues and tumor samples. **F** IHC staining with HAT1 and Pan-Kla on tissue microarray of consecutive sections (scale bar=20 μm). **G** The analysis of correlation between HAT1 and Pan-Kla according to intensity from panel E. **H** The analysis of correlation between HAT1 and Pan-Kla according to IHC score from **F**. All data were analyzed using two-way analysis of variance (ANOVA), *P < 0.05, **P < 0.01.

To investigate whether HAT1 knockout or inhibition using a specific inhibitor enhances radiosensitivity in vivo, we constructed a xenograft model to evaluate radiosensitivity in mice. Results showed that tumor volume and tumor weight decreased in the HAT1-KO group as well as the group treated with HAT1 inhibitor. Moreover, depletion of HAT1 enhanced the radiotherapy sensitivity of mice (Fig. 8A–C). Hematoxylin and eosin (H&E) staining further supported the results mentioned above (Fig. 8D). IHC staining was then performed to assess molecular indicators, including HAT1, Pan-Kla, and Ki-67, and demonstrated that HAT1 knockout inhibits global protein lactylation and and reduces the Ki-67 ratio in mice. (Fig. 8D). To further validate the correlation between HAT1 and pan L-lactyllysine (Pan-Kla) expression in clinical samples, we detected global lactylated proteins and HAT1 expression in 14 pairs of normal and tumor tissues from LUAD patients using western blot. Both HAT1 and Pan-Kla were elevated in tumor samples compared to normal tissue (Fig. 8E), and the expression levels of HAT1 and Pan-Kla showed a significant positive correlation (Fig. 8G). IHC staining of 84 LUAD samples further revealed the positive correlation between HAT1 and Pan-Kla (Fig. 8F, H). These results indicate HAT1 depletion increases radiosensitivity in vivo and suggest the lactyltransferase role of HAT1 in LUAD patients.

## Discussion

The Warburg effect results in the accumulation of lactate within tumor cells [2]. Lactate played various biological effects in tumor cells through metabolic programming and PTM [5–7, 23, 24, 40–42]. Currently, research on the regulatory mechanisms of lactylation is still in its nascent stages, and there are many unresolved questions requiring further investigation. These include identifying the profile of proteins and specific sites that can undergo lactylation, elucidating the effects of lactylation on protein function, and determining which enzymes regulate lactylation. Lactylation is the latest type of novel acylation modification [22]. According to current research, histone acetyltransferases, deacetylases, and their readers are also key regulatory factors of the novel acylation modification [22]. Based on existing studies, the KAT family proteins have been identified as the main writers responsible for lactylation modification [4–6, 22–24]. In this study, by conducting a comprehensive survival analysis of acetyltransferases, deacetylases, and readers, we demonstrated that elevated expression of HAT1 is significantly correlated with adverse clinical outcomes in LUAD patients (Fig. 1A). Furthermore, we established HAT1 as a lactyltransferase that modulates global lactylation levels in LUAD cells (Fig. 2A and M–O).

Previous studies have shown that lactate can promote HR and cause chemoradiotherapy resistance [5, 6]. However, the research on the mechanisms underlying this phenotype has only just begun to scratch the surface. Previous studies has demonstrated that lactylation of MRE11 and NBS1 within the HR complex regulates HR activity. Our findings reveal that RPA1 lactylation also participates in the HR process (Fig. 6), suggesting that lactylation may be broadly involved in HR process. Interestingly, intersection analysis of the gene set from HAT1 co-immunoprecipitation complexes with HR genes revealed that HAT1 may also interact with MRE11 and NBS1 (Fig. 5A). We present two potential hypotheses to explain these findings. The first hypothesis is that lactylation may not follow a one-to-one enzymatic relationship, with multiple enzymes potentially catalyzing lactylation of a single substrate, and HAT1 could function as a lactyltransferase for MRE11 and NBS1. The second hypothesis is that MRE11 and NBS1 may not be direct interactors of HAT1 but could be co-immunoprecipitated indirectly via their association with RPA1, implying that they are not direct substrates of HAT1. Furthermore, the detection of lactylation sites on MRE11 and NBS1 in LUAD cells suggests that HR complex lactylation may have a conserved regulatory role in HR activity across diverse tissues.

Although HAT1 was the first histone acetyltransferase to be identified, its biological function was still not fully revealed. Despite some studies reporting that HAT1 can promote chemotherapy resistance in pancreatic cancer and liver cancer [43, 44], research on its functional roles in tumors is still very limited. At present, whether HAT1 plays a role in radiotherapy resistance remains poorly understood. Recent studies have reported that HAT1 also exhibits succinyltransferase activity [28]. Nevertheless, knockout of HAT1 in LUAD cells did not lead to a significant reduction in succinylation levels (Fig. 2D and Supplementary Fig. S2D), suggesting that the succinyltransferase activity of HAT1 may be tissue-specific, and its underlying mechanisms may require further investigation. Additionally, although HAT1 knockout significantly reduced lysine lactylation levels in LUAD cells, they were not completely abolished (Fig. 2A and Supplementary Fig. S2A), indicating that lysine lactylation may be regulated by multiple enzymes or that non-enzymatic pathways might exist [45]. Noticeably, although the acetylation levels of some proteins were almost completely abolished in HAT1 knockout cells (Fig. 2K and Supplementary Fig. S2K), the molecular weight of high-abundance acetylated proteins appeared to be more concentrated in smaller proteins (below 50 kDa), while high-abundance lactylated proteins (Fig. 2A and Supplementary Fig. S2A), except histones, seemed to be more enriched in larger proteins (above 50 kDa). These findings suggest that the biological functions of acetylated and lactylated proteins may differ. They also imply that the acetyltransferase and lactyltransferase activities of HAT1 may switch based on the size of substrate proteins.

As an ssDNA-binding protein, RPA1 plays a key role in the HR process [17]. It can protect the ssDNA structure formed after DNA end resection and recruit RAD51 to facilitate subsequent HR processes. Previous studies have shown that crotonylation of RPA1 contributes to its HR activity [46]. Besides, although crotonylation and lactylation overlap at the K88 site of RPA1 (Fig. 5B), the other sites lactylated on RPA1 are specific to lactylation, suggesting that different PTMs may work together to enhance the same biological process. This also indicates the widespread participation of different PTMs in the HR process, so whether there are other PTMs involved in HR still needs to be further investigated.

According to previous research, auto-acetylation of KAT family proteins can regulate their acetyltransferase activity [37–39]. Therefore, we examined whether HAT1 has auto-lactylation sites in our lactylome data (Supplementary Data 1) and found that the K15 site of HAT1 can be lactylated (Fig. 7D). We hypothesized that auto-lactylation of HAT1 might regulate its lactyltransferase activity, and further experiments confirmed that K15R mutant of HAT1 impairs its lactyltransferase activity (Fig. 7H). These findings suggest that auto-lactylation of KAT family proteins may serve as a predictor for its lactyltransferase activity. Accordingly, among the four known lactyltransferases in the KAT family (P300, CBP, KAT5, and KAT8), P300 and KAT8 were found to have auto-lactylation sites in our lactylome data (Supplementary Data 1). Although lactylation sites were not detected for CBP and KAT5 in our lactylome data, this could be due to technical limitations in the mass spectrometry process or tissue-specific expression patterns. However, we successfully detected auto-lactylation of all four known lactyltransferases (P300, CBP, KAT5, and KAT8) through immunoprecipitation and western blotting (Fig. 7B). Additionally, although auto-lactylation was not detected in other KAT family proteins using pan-Kla antibodies, we cannot completely exclude the possibility of auto-lactylation and lactyltransferase activity in these proteins due to potential limitations in antibody recognition. Furthermore, for other protein acylation types such as crotonylation, succinylation, and 2-hydroxyisobutyrylation, auto-acylation status may also serve as a potential predictor for the corresponding enzyme activities of KAT family proteins.

In summary, our results suggest for the first time that HAT1 functions as a lactyltransferase and can globally regulate lactylation levels in LUAD cells. HAT1 promotes HR and further contributes to radioresistance through modulating RPA1 lactylation. Our findings provide new evidence for recent studies suggesting that protein lactylation, driven by the Warburg effect, may play a critical role in DNA repair and chemoradiotherapy resistance. Further investigation into the functional roles of protein lactylation in tumor cells will expand our knowledge of how PTMs take part in biological functions and may provide clues for novel therapeutic strategies. Targeting HAT1 may offer a new approach for radiosensitization in LUAD patients.

## Methods and Materials

### Antibodies and reagents

Rabbit reinforced polymer detection system (#PV-9001) and DAB substrate kit (#ZLI-9017) used for IHC were supplied by ZSGB-BIO. Beyotime Biotechnology supplied Crystal violet staining solution (C0121) for the colony formation assay. Comet assay was conducted using Comet assay single cell gel electrophoresis kit (#4250-050-K) from Bio-Techne (Minneapolis, USA). NALA (#1614308) was obtained from Sigma-Aldrich. Lactyl-CoA (#HY-141540) and HAT1 inhibitor JG-2016 (#HY-154944) were purchased from MedChemExpress (MCE). Antibodies against HAT1 (#11432-1-AP), β-actin (#66009-1-Ig), GAPDH (#60004-1-Ig), FLAG (#66008-4-Ig), HA (#51064-2-AP), BLM (#30254-1-AP), MRE11 (#10744-1-AP), NBS1 (#55025-1-AP) were obtained from Proteintech Group. Pan-Kla (#PTM-1401RM), Pan-Kcr (#PTM-502), Pan-Kbu (#PTM-301RM), Pan-Ksu (#PTM-419), Pan-Kglu (#PTM-1152), Pan-Kbz (#PTM-762), Pan-Kpr (#PTM-203), Pan-Kbhb (#PTM-1201RM), Pan-kma (#PTM-902), Pan-Khib (#PTM-802) and Pan-Kac (#PTM-105RM) were purchased from PTM BIO. γ-H2AX (#9718) and RPA1 (#2267) were purchased from Cell Signaling Technology. RAD51 (#PA5-27195) was purchased from Thermo Fisher. Ki67(#HA721115) was obtained from HUABIO. For co-Immunoprecipitation, HA tag (C29F4) rabbit mAb (Magnetic bead conjugate) (#11846S) was purchased from CST. Anti-FLAG® M2 magnetic beads (#M8823) were obtained from Sigma Aldrich. Nano-Traps, including Flag-Trap (ffa) and HA-Trap (atma) for immunoprecipitation with low background, no extra bands, and high specificity, were purchased from Proteintech Group.

### Plasmids and sgRNAs

The sequence targeted to HAT1 used for the construction of lentiviral sgRNA was 5′-GCTACGCTCTTTGCGACCGT -3′. The sequences encoding Flag-tagged HAT1, Flag-tagged RPA1, HA-tagged p300, HA-tagged CBP, HA-tagged HAT1, HA-tagged HAT2A, HA-tagged HAT2B, HA-tagged HAT5, HA-tagged HAT7, HA-tagged HAT8, and His tagged RPA1 were amplified by PCR. PCR fragments were cloned into the pLV-Puro-CMV vector. RPA1 mutants, including K88R, K163R, K167R, and K267R, and HAT1 mutant K15R, were constructed by QuikChange Lightning Site-Directed Mutagenesis Kit.

### Cell culture and transfection

Human lung adenocarcinoma cell lines A549 and H1299 were cultured in RPMI 1640 medium supplemented with 10% fetal bovine serum (FBS) and 1% penicillin/streptomycin (P/S). Dulbecco’s modified Eagle’s medium (DMEM) containing 10% FBS and 1% P/S was used for maintaining HEK-293T cells. All cell lines were cultured in the 5% CO_2_ incubator at 37 °C with saturated humidity. Transfection was performed with Lipofectamine 2000 as described in the manufacturer’s instructions. All cell lines were obtained from ATCC and authenticated by STR profiling.

### IHC staining and scoring

IHC staining was performed to assess the expression of HAT1, pan-Kla, and Ki-67. In detail, samples were incubated in 60 °C oven to melt paraffin, followed by deparaffinization using xylene, and rehydration using gradient ethanol. Citrate buffer was then used for antigen retrieval of tissues. Cell permeabilization was performed with 0.3% TritonX-100. Endogenous peroxidase was then blocked using 3% H2O2. Subsequently, sections were incubated with primary antibodies against HAT1 (1:100), Pan-Kla (1:100), and Ki67 (1:100) at 4°C overnight. The next day, the Rabbit reinforced polymer detection system and DAB substrate kit were used to visualize the samples. Hematoxylin was used for counterstaining. The stained sections were ultimately evaluated by microscopy. Tissue sections were quantitatively evaluated using a dual-parameter scoring system assessing both the percentage of immunopositive cells and staining intensity. Proportion scoring was defined as follows: No positive staining (0% of tumor cells); 1.0: 0.1–1% positive cells; 2.0: 1.1–10% positive cells; 3.0: 11–30% positive cells; 4.0: 31–70% positive cells; 5.0: 71–100% positive cells. Staining intensity was graded on a 4-tier scale: 0: Negative; 1: Weak; 2: Moderate; 3: Strong. The composite score (range 0-8) was derived by summing the proportion and intensity scores.

### Western blot

Tissues and cells were collected and lysed in RIPA buffer (50 mM Tris pH = 7.4, 150 mM NaCl, 1% Triton X-100, 1% sodium deoxycholate, and 0.1% SDS) supplemented with Phenylmethanesulfonyl fluoride (PMSF). Following quantification and denaturation of protein, whole protein lysates were separated using SDS-PAGE gels and then transferred to PVDF membranes. The membranes were blocked in 5% skim milk and then incubated with primary antibodies at 4 °C overnight. The following day, the membranes were washed and incubated with horseradish peroxidase (HRP) conjugated secondary antibodies. Proteins on membranes were ultimately visualized by ECL reagents and then captured by the ChemiDoc MP system.

### Immunofluorescence

To investigate the foci of γ-H2AX, RPA1, and RAD51, Immunofluorescence was conducted in A549 and H1299 cells. In brief, cells were counted and seeded onto cover slips in 12-well plates. After attaching overnight, cells were treated with 8 Gy Irradiation. Cells were fixed with 4% paraformaldehyde (PFA) at the indicated time points, including 0 h, 1 h, 4 h, and 24 h. Fixed cells were permeabilized with 0.3% Triton X-100 and then blocked with 10% goat serum, incubated with indicated primary antibodies against γ-H2AX (1:200), RPA1 (1:50), and RAD51 (1:100) at 4 °C overnight. The next day, cells were incubated with fluorescence-linked secondary antibodies. The cover slips were sealed with an anti-fade reagent containing DAPI. The foci of γ-H2AX, RPA1, and RAD51 in random fields were observed by a Leica Stellaris 5 confocal fluorescence microscope.

### Comet assay

Comet assay was performed to assess the DNA damage in A549 and H1299 cells. Specifically, cells cultured in 60 mm dishes were treated with 8 Gy IR. Cells were harvested at the indicated time points. Counting and diluting cells into 1 × 10^6^ cells /ml, cells were gently mixed with molten LMAgarose at a ratio of 1:10 (V/V), mixture was added onto the comet slides immediately. Following gelling, the slides were incubated with lysis buffer at 4 °C overnight. The next day, the slides were subjected to electrophoresis at 21 V for 30 min. Before observing by fluorescence microscopy, cells were stained with Nucleic acid dye goldview (1:10000). The tail moment was analysed by the Comet Assay Software Project (CASP).

### Colony formation assay

For the colony formation assay, A549 and H1299 cells were counted and seeded in 6-well plates (1000 cells/well). Cells were then treated with various doses of IR, including 0 Gy, 2 Gy, 4 Gy, 6 Gy, and 8 Gy. Irradiated cells were cultured in standard conditions for about 1–2 weeks. When obvious colonies were observed, the colonies were fixed with 4% PFA and stained with crystal violet. The colonies were captured and counted.

### HR and NHEJ reporter assay

DR-GFP reporter systems were used to monitor HR efficiency, and EJ5-GFP reporter systems were used to detect NHEJ efficiency. Briefly, cells were transfected with DR-GFP and I-SceI plasmids using Lipofectamine 2000 or with EJ5-GFP and I-SceI plasmids. After 48 h, cells were collected and performed flow cytometry analysis, sorting by GFP-positive cells, and the ratios of HR efficiency or NHEJ efficiency were calculated.

### 4D label-free lactylation quantitative proteomics and analysis

4D label-free lactylation proteomics in A549 cells was performed by PTM BioLab.

### Immunoprecipitation

Cells were washed with cold PBS and Scrape cells into cold PBS. Cells were then lysed using Western and IP lysis buffer on ice for 30 min, centrifuged cells at 12,000 rpm for 30 min at 4 °C. Remove supernatant to a new tube and transfer a 50 μL sample of whole cell lysates to a new tube as input. Whole cell lysates were then pre-cleaned with corresponding protein A or protein G magnetic beads for 1 h at 4 °C with rotation. For each IP sample, transfer supernatant to a new tube, add magnetic beads conjugated antibodies against IgG, Flag, HA, and RPA1 to the IP sample, and incubate at 4 °Covernight with rotation. The following day, the magnetic beads were washed and denatured at 95 °C using 1x SDS sample buffer to elute proteins from the magnetic beads for 10 min. The expression of RPA1, HAT1, Pan- Kla and GAPDH was detected by Western blot. Nano-Traps from Proteintech Group, including Flag-Trap (ffa) and HA-Trap (atma), were used for immunoprecipitation of the target band, which may overlap with IgG, demonstrating low background, absence of extra bands, and high specificity.

### Molecular docking

Molecular docking was performed to assess the binding affinity between HAT1 and La-CoA. The structure of HAT1 (Alphafold ID: AF-O14929) was obtained from the Alphafold website (https://alphafold.ebi.ac.uk/entry/O14929). The La-CoA data were obtained from the PubChem database (https://pubchem.ncbi.nlm.nih.gov/) to download the SDF file. AutoDock vina (version 2.0) was used to perform the docking of HAT1 with La-CoA. The docking pose with the lowest binding energy (representing the highest binding affinity) was selected as the final docking result. The docking results were visualized by PyMoL software.

### In vitro lactylation assay

HA-tagged HAT1 proteins were purified from HEK293T cells and then added in the reaction buffer (50 mM HEPES, pH7.8, 30 mM KCl, 0.25 mM EDTA, 5.0 mM MgCl2, 5.0 mM sodium butyrate, 2.5 mM DTT). The His fusion RPA1 proteins were incubated with HA-tagged HAT1 proteins at 30 °C for 30 min with 20 mM lactyl-CoA, followed by adding 5 × SDS sample buffer and heating at 95 °C for 5 min to stop the reaction. Pan-Kla, Pan-ac, HAT1, and RPA1 were detected by Western blot.

### DNA-protein binding assay

For the DNA-protein binding assay, biotin-labeled ssDNA was synthesized. HEK-293T cells were transfected with plasmids encoding FLAG-tagged RPA1 or RPA1 mutants, including K88R, K163R, K167R, and K267R. Cells were collected and lysed in binding buffer (10 mM Tris-HCl, PH 7.5, 100 mM NaCl, 10 g/ml BSA, 10% glycerol, and 0.5% NP-40). Whole cell lysates were then incubated with biotin-conjugated ssDNA at room temperature for 30 min. Streptavidin beads (S beads) were added to the ssDNA-lysates mixture at room temperature for 1 h to pull down the proteins interacting with ssDNA. The ssDNA binding proteins were analyzed by Western blot.

### Xenograft model in vivo

All animal studies were conducted in accordance with the relevant guidelines of the National Institutes of Health Guidelines for animal welfare. 4-week-old female BALB/c nude mice were purchased and housed in special pathogen-free (SPF) conditions. A total number of 1 × 10^6^ cells in 100 μL serum-free culture medium were injected subcutaneously into the flanks of mice. When subcutaneous tumors were observed, each group of mice was divided into 2 groups randomly, tumor sites of one group of mice were treated with 5 Gy IR twice, with shielding of the non-tumor areas. Tumor size was measured every 2 days. After death or euthanasia, the tumors were separated carefully, fixed in 4% PFA, and embedded in paraffin. Tumor sections were performed with H&E staining and IHC assay, which detected the expression of indicated proteins, including HAT1, Pan-Kla, and Ki-67. Based on previous studies, ten mice were allocated to each group. The blinding procedure was not performed in the animal study.

### Statistical analysis(Quantification and statistical analysis)

GraphPad software was used for statistical analyses. Each experiment was performed in triplicate at least, and the data were presented as the mean ± SD. Significant differences were calculated by two-way analysis of variance (ANOVA) or Student’s *t* test. *P*-values of < 0.05 (*) and *P*-values of < 0.01 (**) were considered statistically significant.

## Supplementary information

Supplementary information

Supplementary Table 1

Supplementary Table 2

Original western blots

## Data Availability

The lactylation proteomics data can be obtained via Pride (http://www.ebi.ac.uk/pride) with the dataset identifier PXD054919 and will be available publicly upon acceptance. All data are available in the main text or the supplementary materials. Additional data related to this paper may be requested from the authors.
