# Supplementary material for: HAT1 functions as a lactyltransferase and mediates RPA1 lactylation to promote DNA repair and radioresistance in lung adenocarcinoma
Source: Cell Death Dis. 2025 Nov 21;16(1):851. doi: 10.1038/s41419-025-08113-x (PMC12639135; doi:10.1038/s41419-025-08113-x)
Supplement: Supplementary file 1 — Supplementary information [file 41419_2025_8113_MOESM1_ESM.docx]

**HAT1 functions as a lactyltransferase and mediates RPA1 lactylation to promote DNA repair and radioresistance in lung adenocarcinoma**

**Supplementary Information**


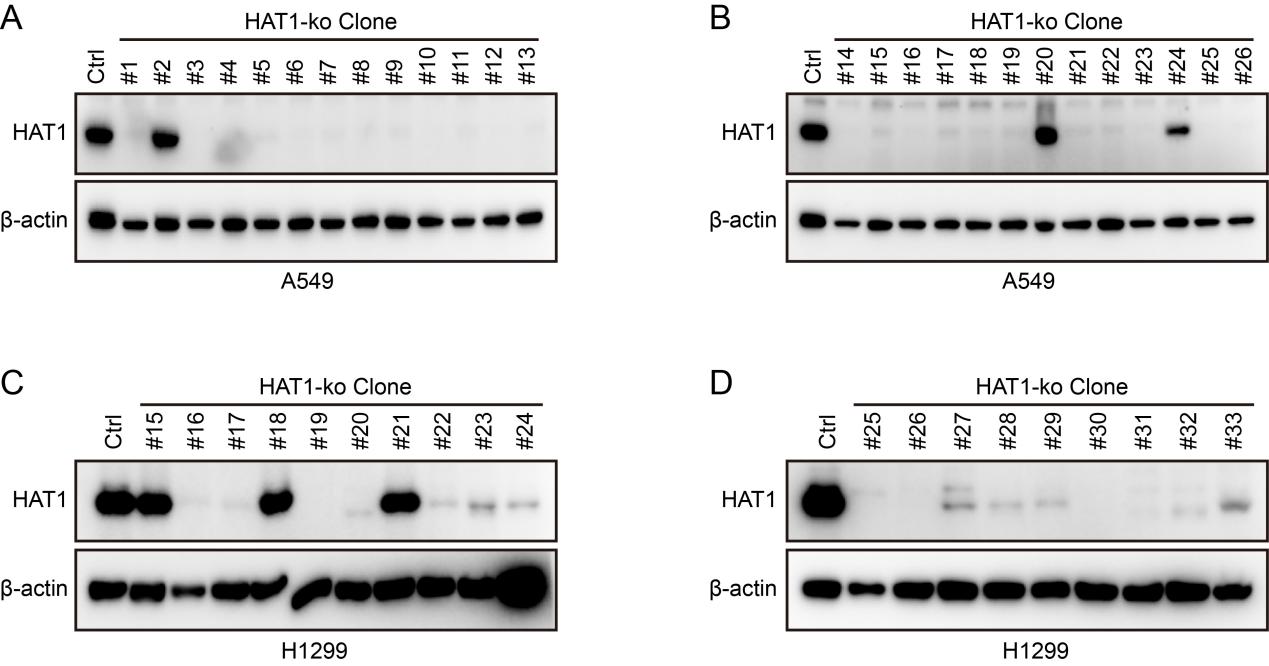


Supplementary Fig. 1 Identification of HAT1 knockout monoclonal. (A) Identification of HAT1 knockout monoclonal in A549 cells. (B) Identification of HAT1 knockout monoclonal in A549 cells. (C) Identification of HAT1 knockout monoclonal in H1299 cells. (D) Identification of HAT1 knockout monoclonal in H1299 cells.


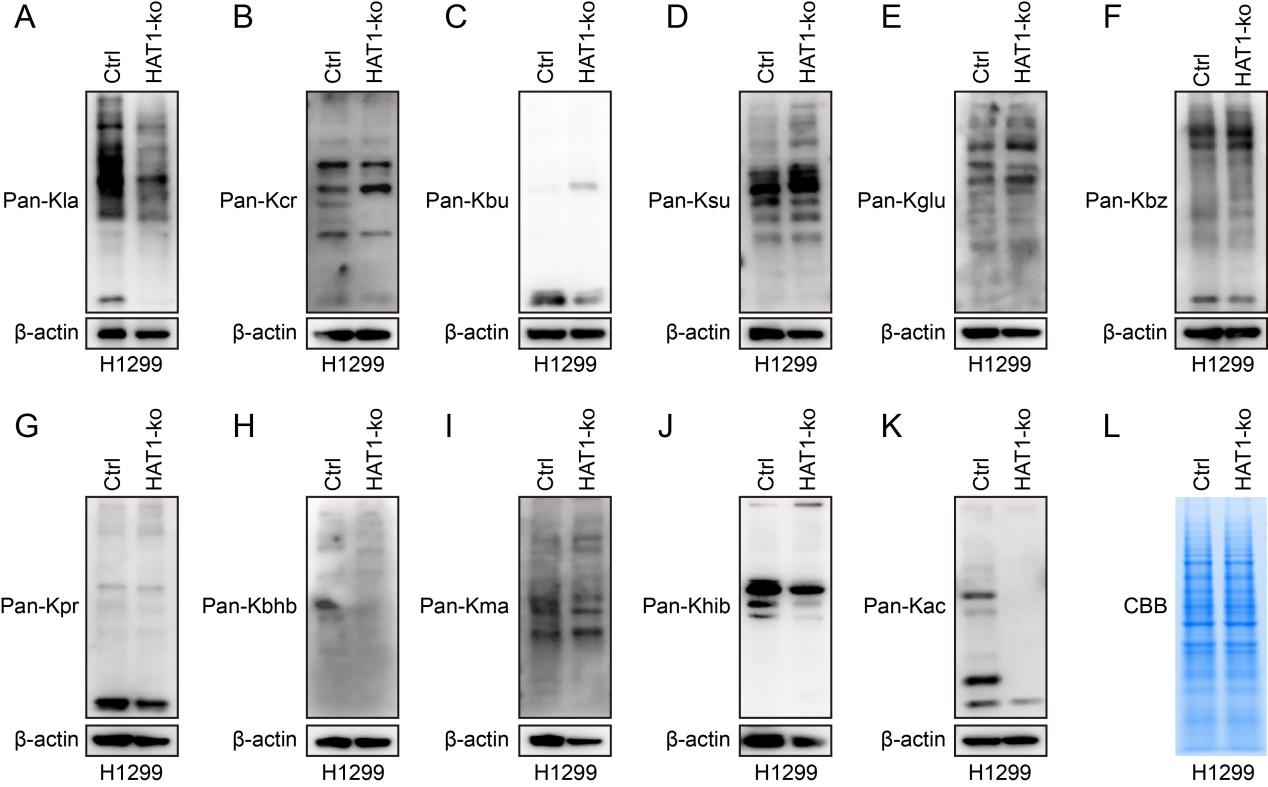


Supplementary Fig. 2 Detection of various acylation modifications in HAT1 knockout H1299 cells. (**A**) The level of pan L-Lactyl Lysine (Pan-Kla) in HAT1-KO Cells. (**B**) The expression of pan Crotonyllysine (Pan-Kcr) in HAT1-KO Cells. (**C**) The level of pan Butyryllysine (Pan-Kbu) in HAT1-KO Cells. (**D**) The expression of global Succinyllysine (Pan-Ksu) in HAT1-KO Cells. (**E**) The level of global Glutaryllysine (Pan-Kglu) in HAT1-KO Cells. (**F**) The expression of global Benzoyllysine (Pan-Kbz) in HAT1-KO Cells. (**G**) The level of pan Propionyllysine (Pan-Kpr) in HAT1-KO Cells. (**H**) The expression of global β-Hydroxybutyryllysine (Pan-Kbhb) in HAT1-KO Cells. (**I**) The level of global Malonyllysine (Pan-Kma) in HAT1-KO Cells. (**J**) The level of pan 2-Hydroxyisobutyryllysine (Pan-Khib) in HAT1-KO Cells. (**K**) The expression of global Acetyllysine (Pan-Kac) in HAT1-KO Cells. (**L**) Coomassie brilliant blue staining of total proteins in control cells and in HAT1-KO cells.


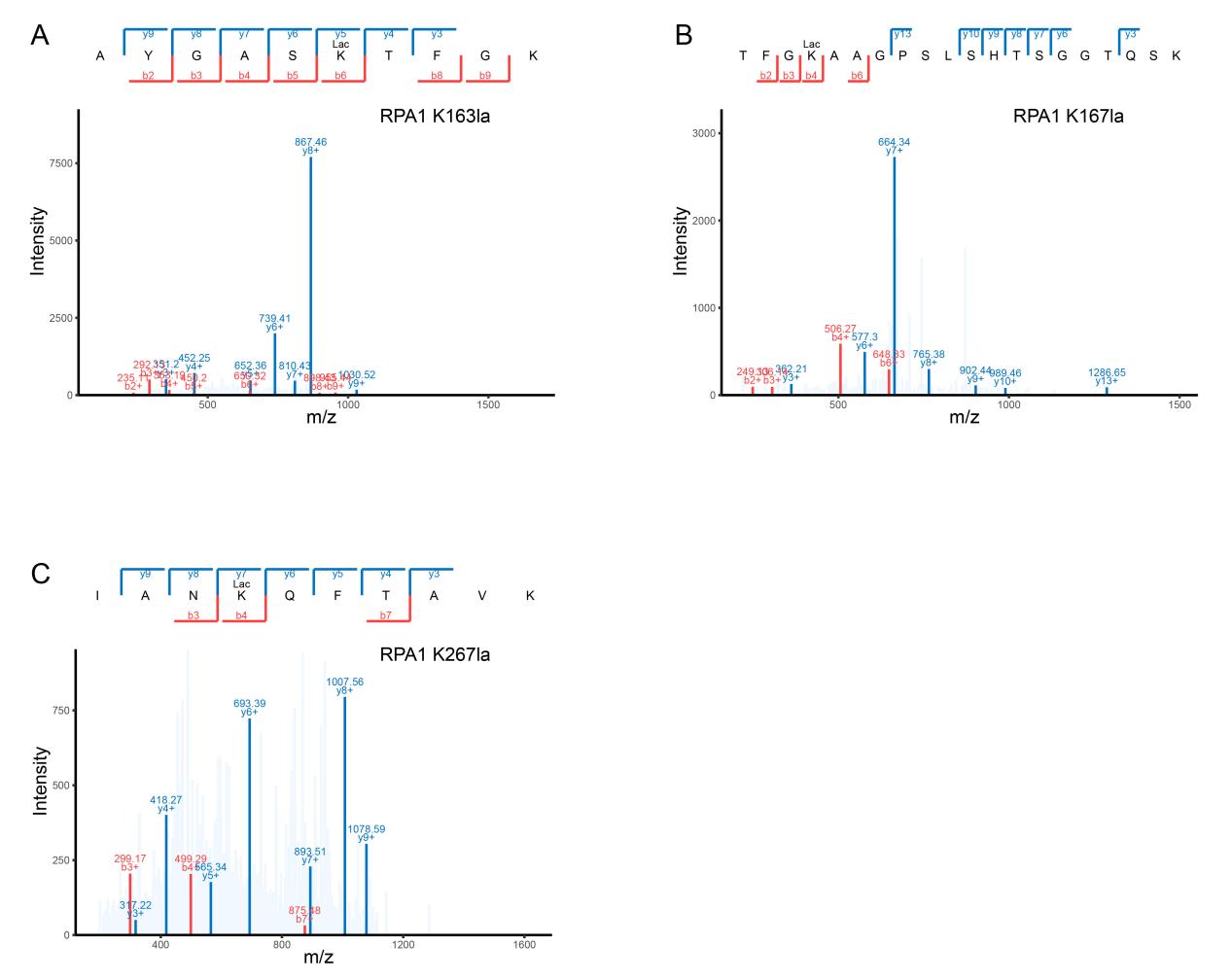


Supplementary Fig. 3 Secondary mass spectra of lactylation at RPA1 K163, K167, and K267 sites. (A) Identification of K163 lactylation of RPA1. (B) Identification of K167 lactylation of RPA1. (C) Identification of K267 lactylation of RPA1.
