# Supplementary material for: HAT1 functions as a lactyltransferase and mediates RPA1 lactylation to promote DNA repair and radioresistance in lung adenocarcinoma
Source: Cell Death Dis. 2025 Nov 21;16(1):851. doi: 10.1038/s41419-025-08113-x (PMC12639135; doi:10.1038/s41419-025-08113-x)

Fig. 1

Figure 1H

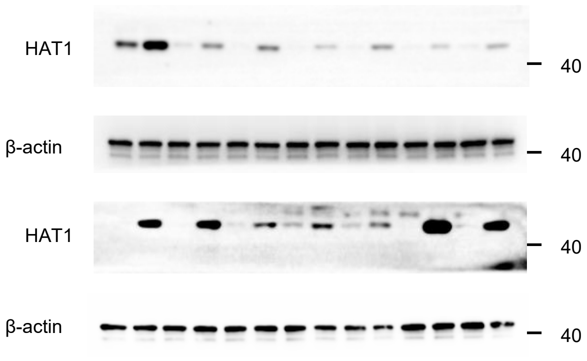

Fig. 2

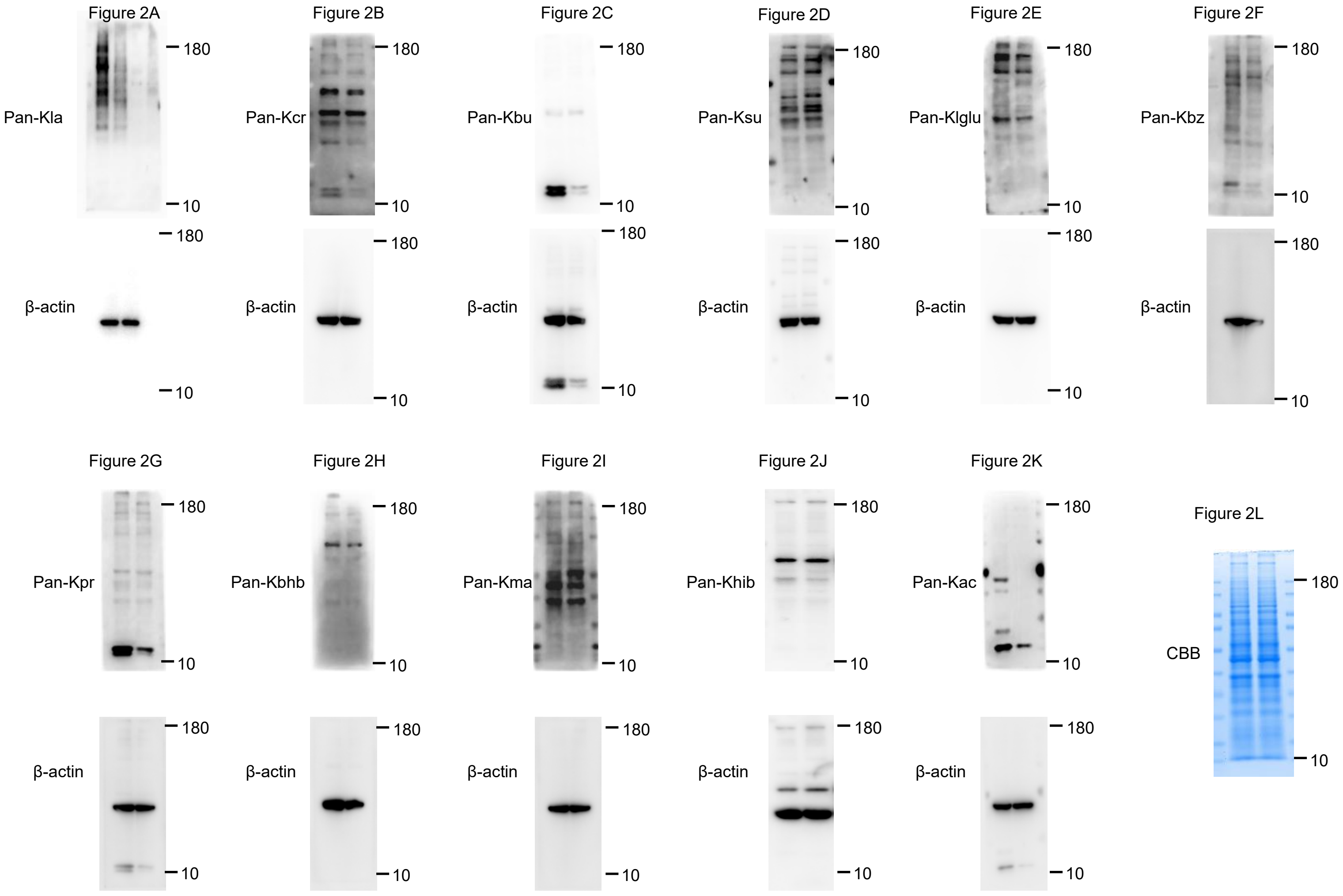
Figure 2A
Pan-Kla
β-actin
Figure 2B
Pan-Kcr
β-actin
Figure 2C
Pan-Kbu
β-actin
Figure 2D
Pan-Ksu
β-actin
Figure 2E
Pan-Klglu
β-actin
Figure 2F
Pan-Kbz
β-actin
Figure 2G
Pan-Kpr
β-actin
Figure 2H
Pan-Kbhb
β-actin
Figure 2I
Pan-Kma
β-actin
Figure 2J
Pan-Khib
β-actin
Figure 2K
Pan-Kac
β-actin
Figure 2L
CBB
180
10

Figure 2M

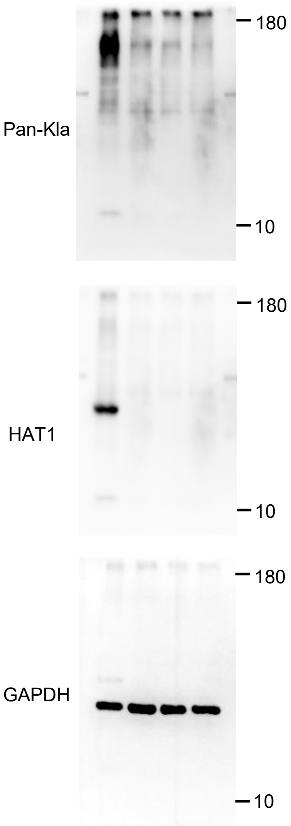

Figure 2N

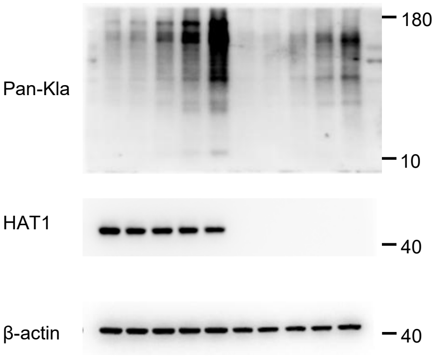

Figure 2O

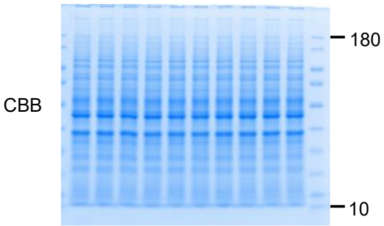

Fig. 3

Figure 3E

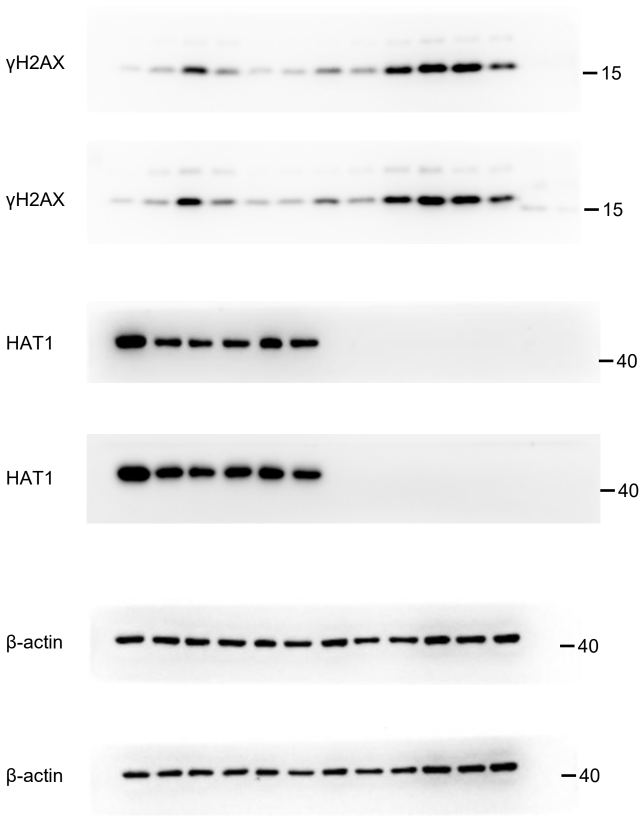

Figure 3F

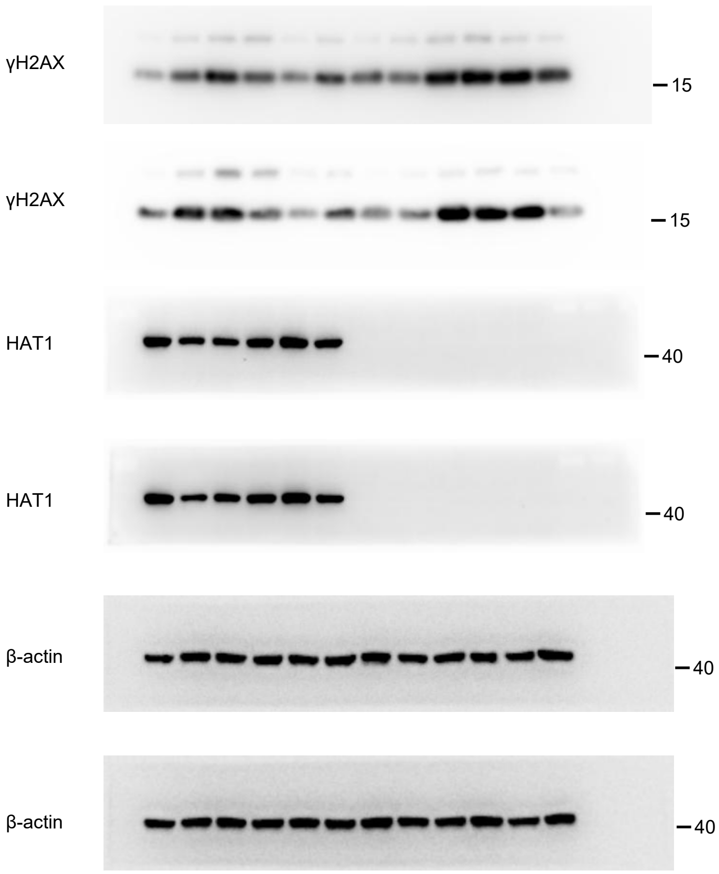

Fig. 5

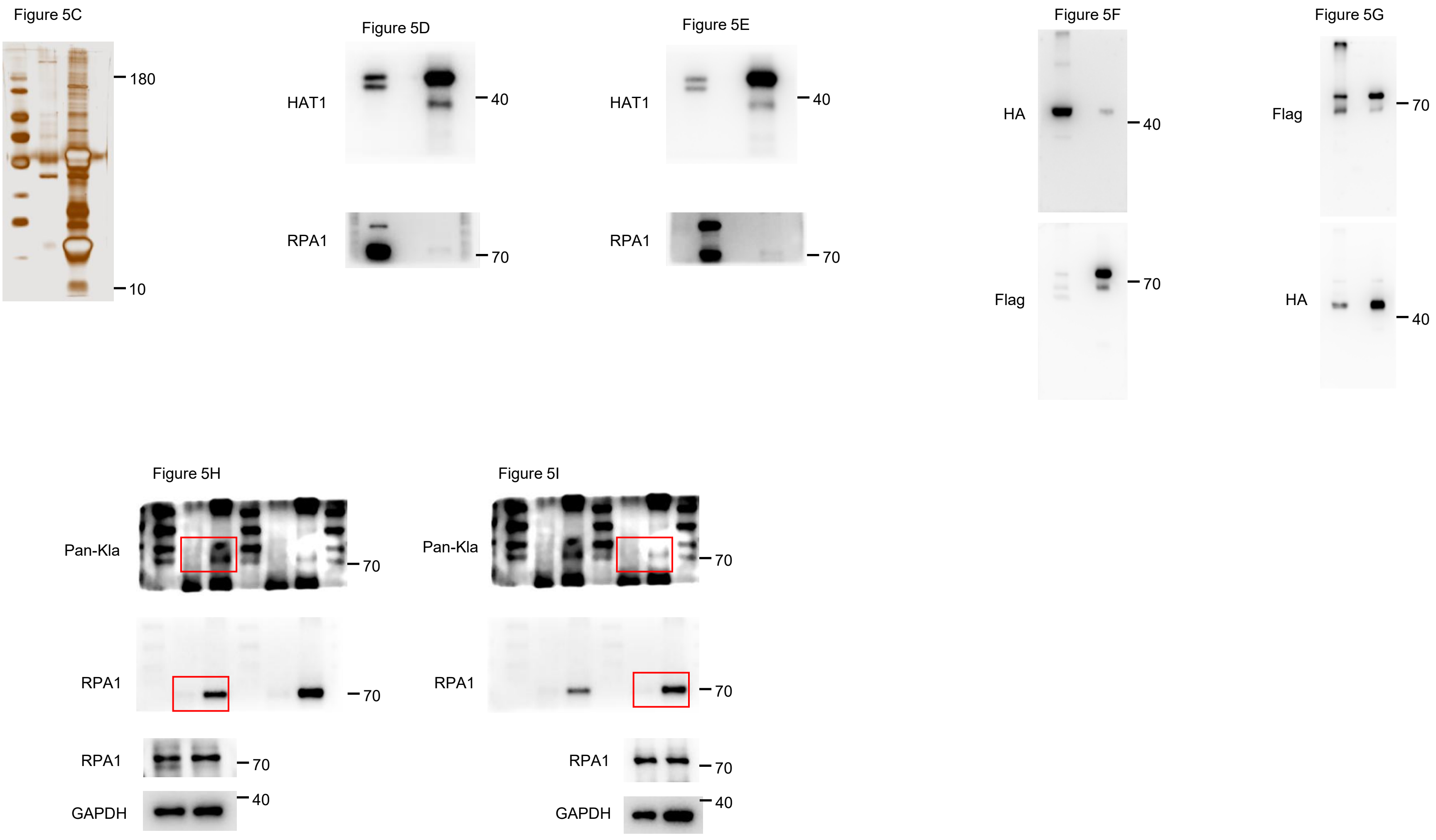
Figure 5C
180
10
Figure 5D
HAT1
40
RPA1
70
Figure 5E
HAT1
40
RPA1
70
Figure 5F
HA
40
Flag
70
Figure 5G
Flag
70
HA
40
Figure 5H
Pan-Kla
70
RPA1
70
RPA1
70
GAPDH
40
Figure 5I
Pan-Kla
70
RPA1
70
RPA1
70
GAPDH
40

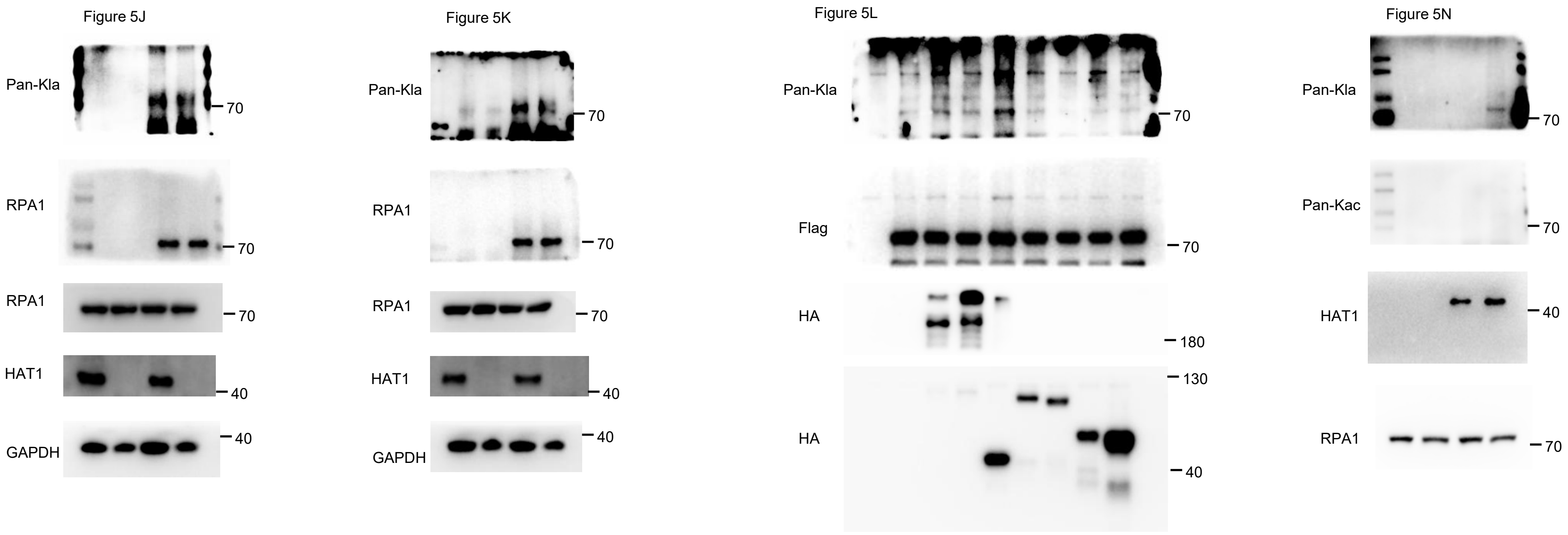

Figure 5J
Pan-Kla
70
RPA1
70
RPA1
70
HAT1
40
GAPDH
40
Figure 5K
Pan-Kla
70
RPA1
70
RPA1
70
HAT1
40
GAPDH
40
Figure 5L
Pan-Kla
70
Flag
70
HA
180
130
HA
40
Figure 5N
Pan-Kla
70
Pan-Kac
70
HAT1
40
RPA1
70

Fig. 6

Figure 6A

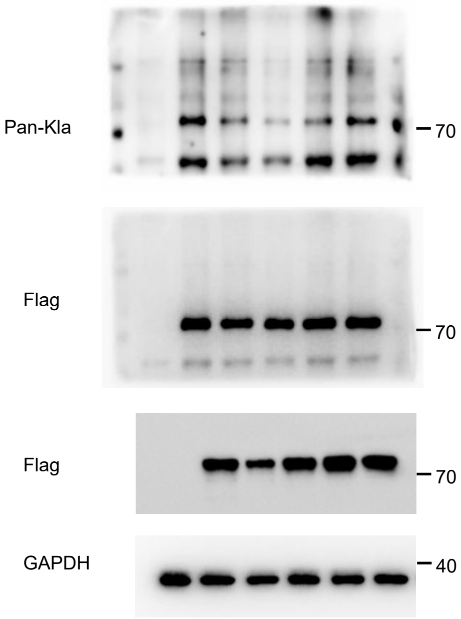

Figure 6B

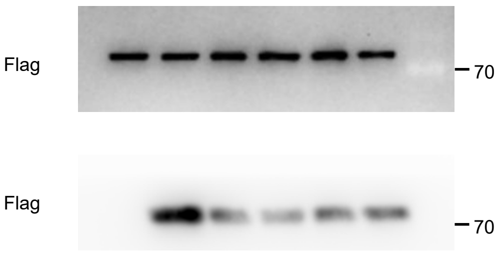

Figure 6C

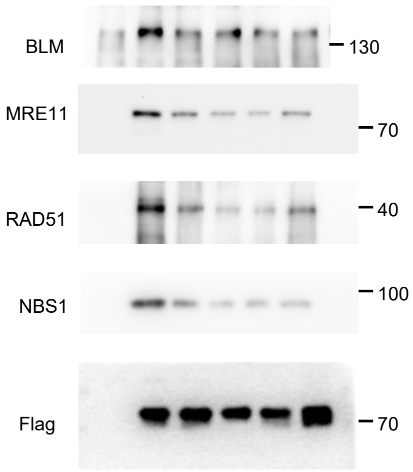

Fig. 7

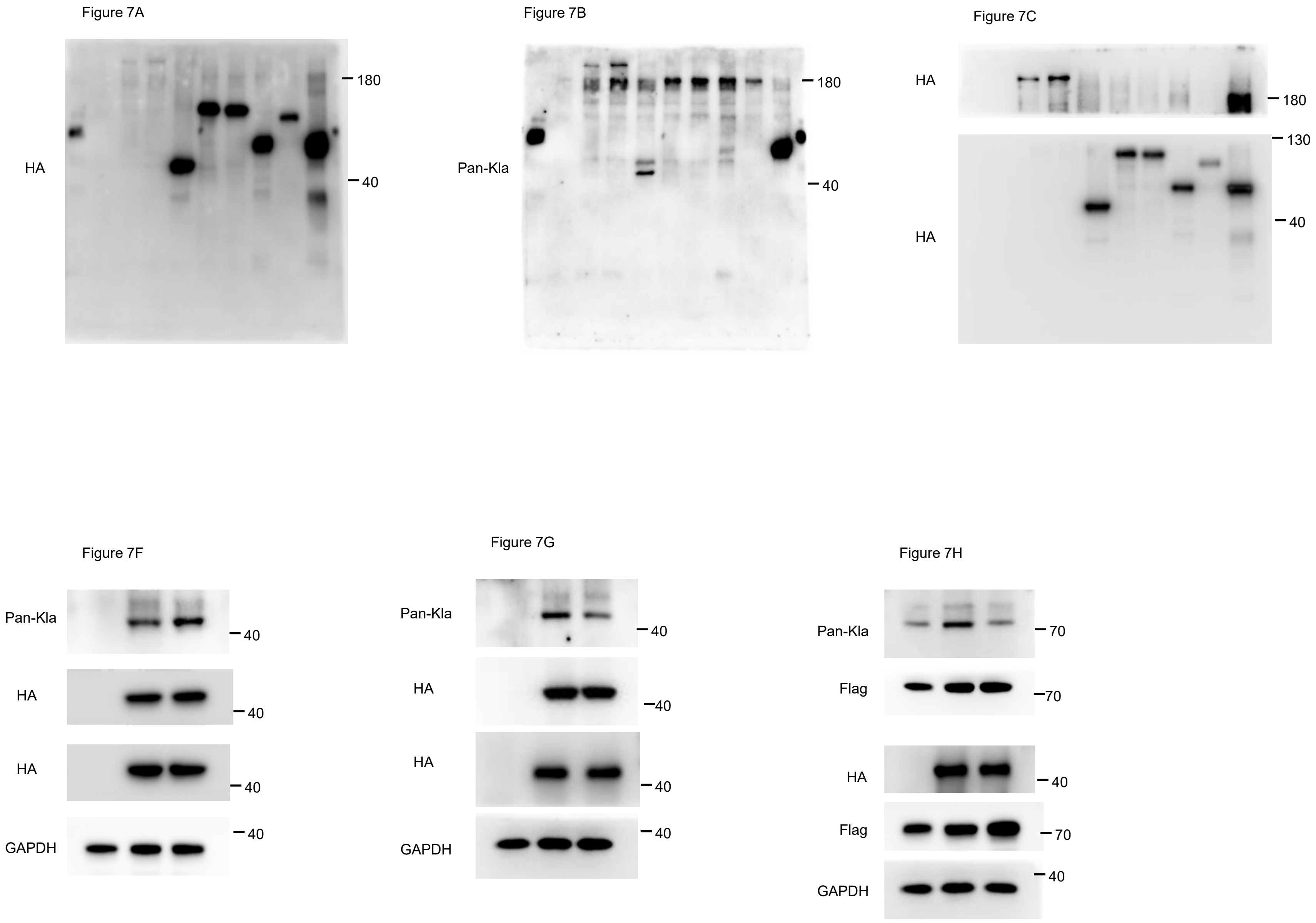

Figure 7A
HA
180
40
Figure 7B
Pan-Kla
180
40
Figure 7C
HA
180
130
HA
40
Figure 7F
Pan-Kla
40
HA
40
HA
40
GAPDH
40
Figure 7G
Pan-Kla
40
HA
40
HA
40
GAPDH
40
Figure 7H
Pan-Kla
70
Flag
70
HA
40
Flag
70
GAPDH
40

Fig.8

Figure 8E

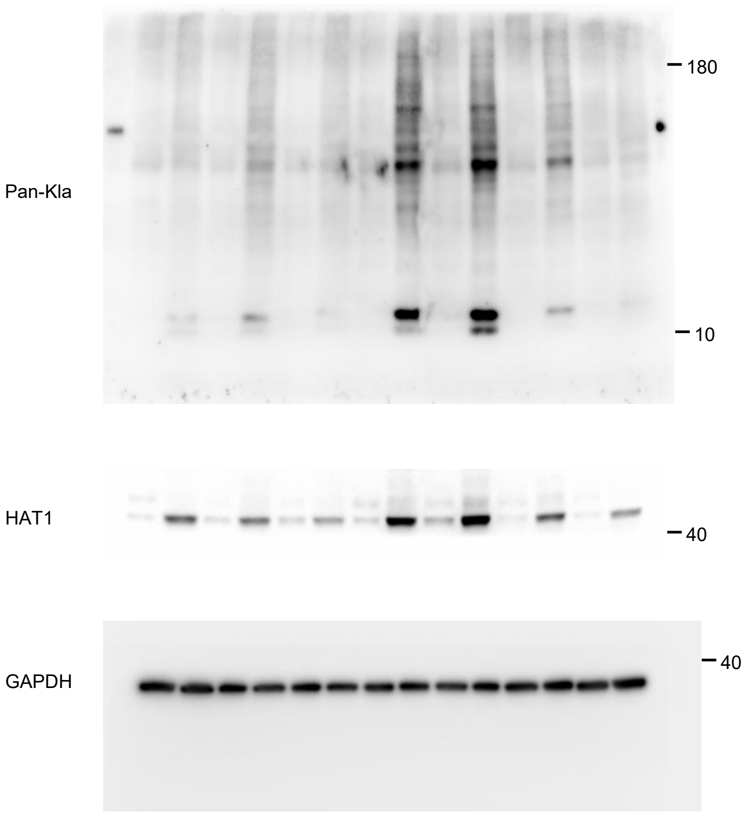

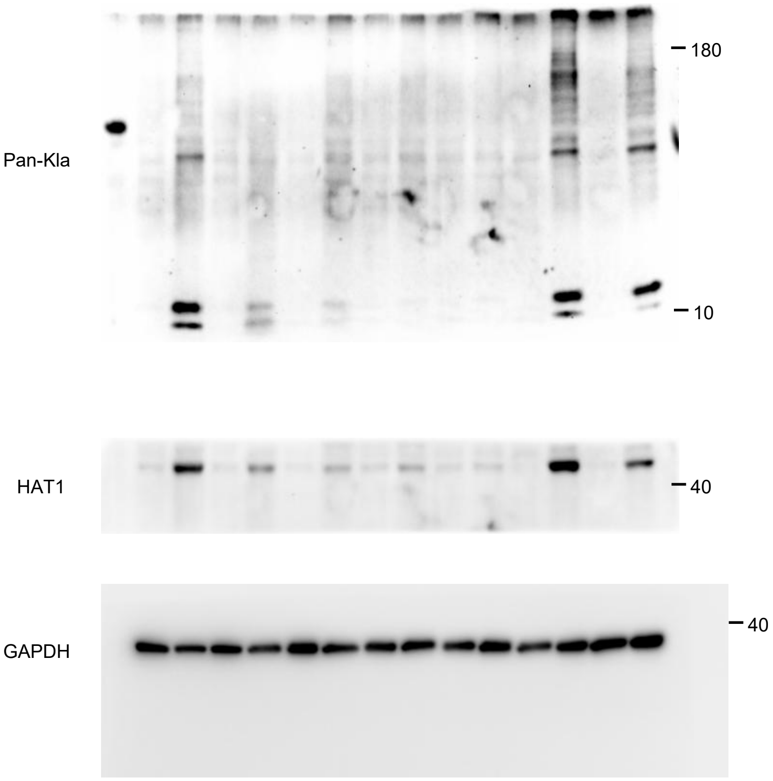

Supplementary Fig. 1

Supplementary Fig 1A

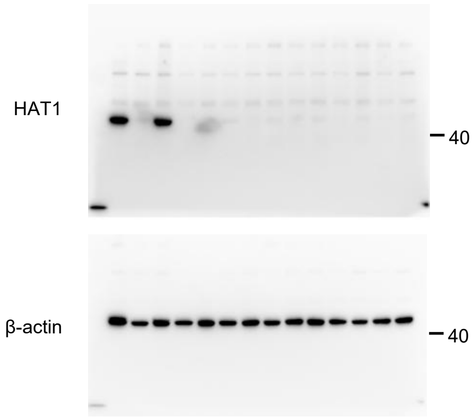

Supplementary Fig 1B

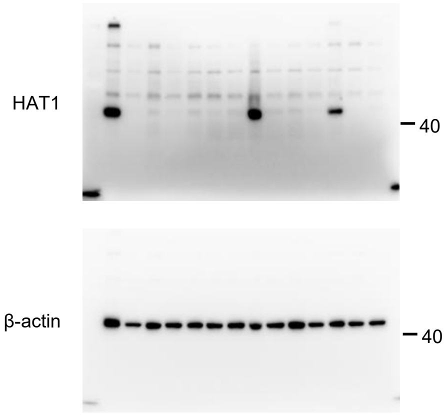

Supplementary Fig 1C

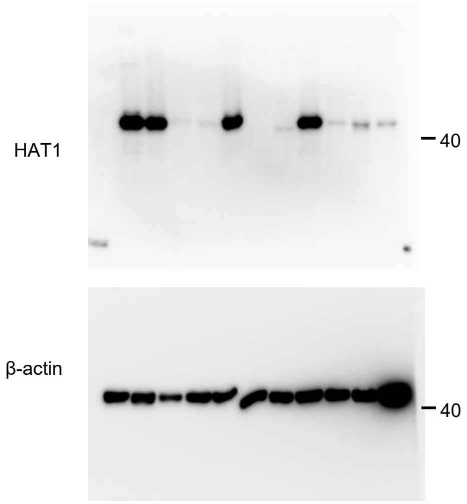

Supplementary Fig 1D

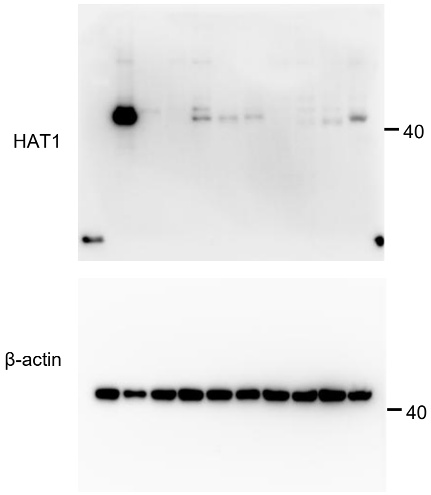

Supplementary Fig. 2

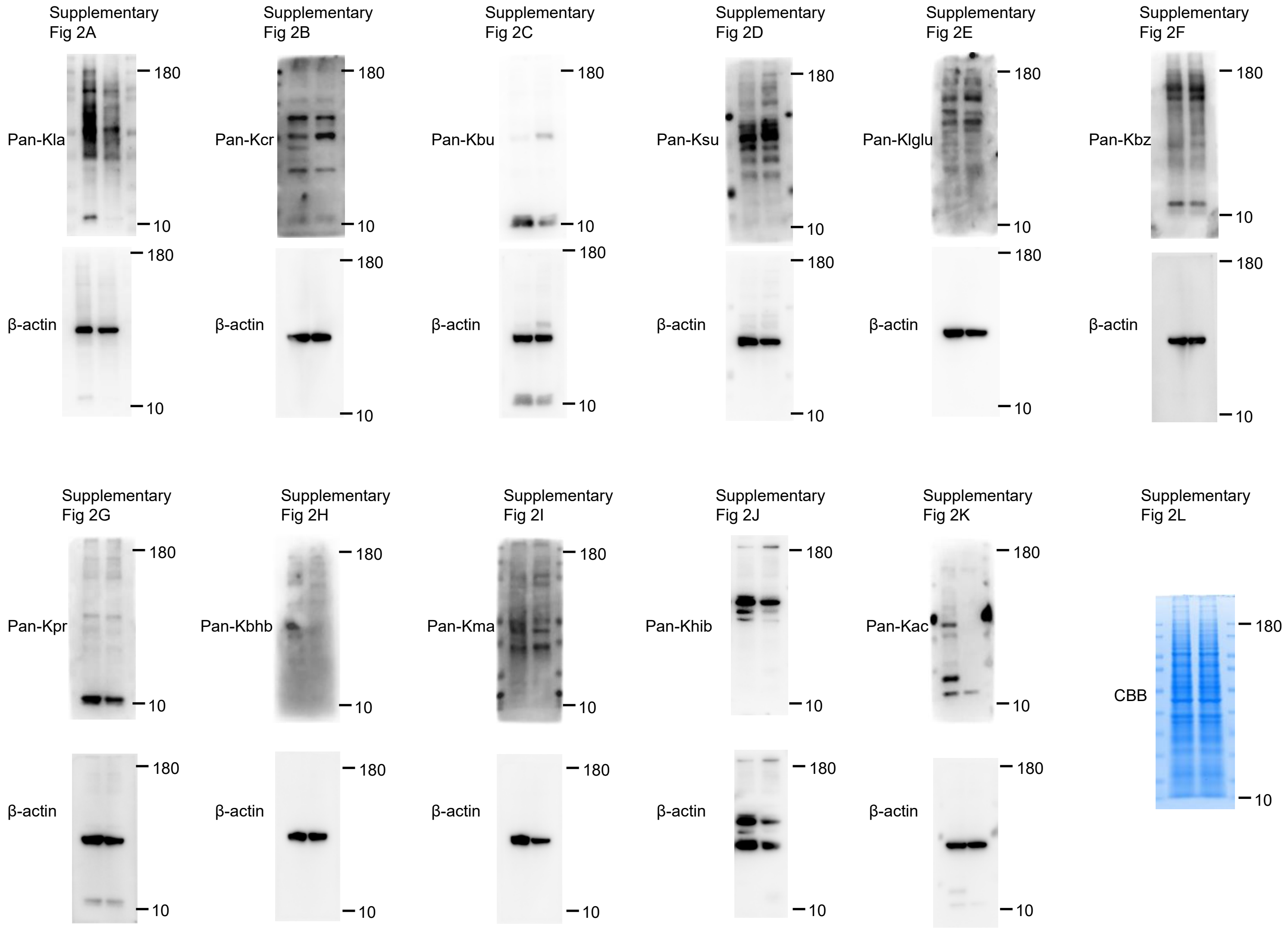

Supplement: Supplementary file 4 — Original western blots [file 41419_2025_8113_MOESM4_ESM.pdf]
